# Designing optimal allocations for cancer screening using queuing network models

**DOI:** 10.1371/journal.pcbi.1010179

**Published:** 2022-05-27

**Authors:** Justin Dean, Evan Goldberg, Franziska Michor

**Affiliations:** 1 Department of Data Science, Dana-Farber Cancer Institute, Boston, Massachusetts, United States of America; 2 Department of Biostatistics, Harvard T. H. Chan School of Public Health, Boston, Massachusetts, United States of America; 3 Department of Stem Cell and Regenerative Biology, Harvard University, Cambridge, Massachusetts, United States of America; 4 Center for Cancer Evolution, Dana-Farber Cancer Institute, Boston, Massachusetts, United States of America; 5 The Broad Institute of MIT and Harvard, Cambridge, Massachusetts, United States of America; 6 The Ludwig Center at Harvard, Boston, Massachusetts, United States of America; Pázmány Péter Catholic University: Pazmany Peter Katolikus Egyetem, HUNGARY

## Abstract

Cancer is one of the leading causes of death, but mortality can be reduced by detecting tumors earlier so that treatment is initiated at a less aggressive stage. The tradeoff between costs associated with screening and its benefit makes the decision of whom to screen and when a challenge. To enable comparisons across screening strategies for any cancer type, we demonstrate a mathematical modeling platform based on the theory of queuing networks designed for quantifying the benefits of screening strategies. Our methodology can be used to design optimal screening protocols and to estimate their benefits for specific patient populations. Our method is amenable to exact analysis, thus circumventing the need for simulations, and is capable of exactly quantifying outcomes given variability in the age of diagnosis, rate of progression, and screening sensitivity and intervention outcomes. We demonstrate the power of this methodology by applying it to data from the Surveillance, Epidemiology and End Results (SEER) program. Our approach estimates the benefits that various novel screening programs would confer to different patient populations, thus enabling us to formulate an optimal screening allocation and quantify its potential effects for any cancer type and intervention.

## Author summary

We describe a mathematical modeling methodology that offers quantitative insights into the potential benefits of screening and other interventions on cancer mortality. Our queuing-theoretic approach represents a potentially useful alternative to more traditional modeling approaches, in that it can provide more detailed results and entirely circumvents the need for simulations. Our methodology can be widely applied to estimate costs and benefits of screening strategies. By providing a detailed example of our method applied to epidemiological data, we hope to encourage greater uptake of this methodology in the community.

This is a *PLOS Computational Biology* Methods paper.

## Introduction

Cancer is a potentially fatal disease with a large annual incidence worldwide [1]. Since it is the result of the gradual accumulation of genetic and/or epigenetic changes [2] that eventually lead to uncontrolled proliferation and dissemination of cells, its stage at diagnosis has a large impact on a patient’s prognosis [3]. Therefore, diagnosing cancer early through screening can result in substantially reduced mortality and treatment-associated morbidity [4]. For most cancer types, sensitive screens remain unavailable [5] and even in cases when screening technology exists, screens take time, are expensive, and often lead to psychological distress [6], particularly regarding false positives and possibly overtreatment [7]. In some cases, screening has not been demonstrated to prolong survival, for instance with PSA screening for prostate cancer [8]. These tradeoffs lead to considerations regarding the costs and benefits of different screening programs. The advent of novel diagnostic tools that can detect signatures of circulating tumor DNA (ctDNA) in plasma heralds a revolution in early cancer detection [9],[10],[11],[12],[13]. Using these assays, mutations or epigenetic states of interest can be characterized without the need for an invasive biopsy. Innovations such as these advances might make previously unviable cancer screening programs soon worth pursuing on a more widespread basis, motivating the development of mathematical models of such potential screening programs and their optimization based on incidence and survival data.

Quantifying the costs and benefits of screening strategies is necessary for identifying optimum approaches. Many mathematical modeling approaches for designing screening protocols use ordinary differential equations (i.e., compartmental models) or Markov chains. For instance, Yaffe et al [14] employ a microsimulation model of mammography screening to compare the efficacy and cost effectiveness of various breast cancer screening programs. Similarly, Mandelblatt et al [15] use a combination of different simulation models to determine optimal breast cancer screening strategies, predicting whom to screen and how often. In Altrock et al [16], we develop a simulation approach to determine the effectiveness of screening schedules for patients with monoclonal gammopathy of undetermined significance (MGUS), which are at an increased risk for progressing to multiple myeloma (MM). Kobayashi et al [17] use a Markov model to determine optimal intervals between prostate cancer screens based upon measurements of prostate specific antigen (PSA). Underwood et al [18] use a stochastic simulation for PSA-threshold based prostate cancer screening to identify the best policy in terms of maximizing quality-adjusted life years (QALYs). Similarly, Chen et al [19] determine the optimal age of performing colonoscopies for colorectal cancer screening using a Markov model. Berger et al [20] develop a clinical effectiveness model of a fecal-based DNA test that projects incidence and mortality of colorectal cancer under different intertest intervals using a 5-arm in silico clinical trial. These examples serve as illustrations of various mathematical modeling approaches for designing and assessing screening programs.

A disadvantage of systems of ODEs is that they are continuous and deterministic, whereas the populations and state changes they model are discrete and stochastic since phenomena such as developing a disease are inherently random. When considering only average quantities of large populations, stochastic models offer little extra over their deterministic counterparts. However, when populations are small (as in the case of a rare disease or a particular population subgroup), or considering metrics that go beyond mere averages, such as the variance or tail probabilities of a certain outcome, then stochastic models offer additional utility. Markov chain models assume exponential waiting times, implying that processes that they model are memoryless, with constant hazard rates. These are very stringent modeling assumptions made for mathematical simplicity, but they are unrealistic in many settings. For instance, knowing how long a patient has lived may be very informative about their residual lifetime. These shortcomings of existing methodology lead us to hypothesize that the theory of queuing networks [21] may be useful for designing improved approaches.

Queuing networks are discrete-valued stochastic processes that track the time evolution of populations of agents. Unlike Markov chains, they do not necessarily assume exponential waiting times but can be analyzed in a very general setting, yielding analytical expressions of the full joint stationary probability distribution of the network. Such distributional results are useful for predicting fluctuations in demand—something deterministic models cannot do. These results can be used to forecast resource allocation such as staffing levels, number of hospital beds, and others, or the number of insurance claims that will be made which helps when setting premiums or budgeting government resources, both of which depend not only on averages but on the whole distribution. Additionally, operational laws provide qualitative closed-form expressions for model outputs in terms of inputs, whereas pure simulation models yield merely quantitative descriptions (see the Results section for a more in-depth comparison).

Several applications of queuing-theoretic models to healthcare have been developed. Green [22] use finite server queues to determine the capacity levels of staffing and beds in hospitals to address the fundamental tradeoff between delay reduction and redundancy. A finite server queue is one in which there is dependence between different agents’ waiting times due to sharing of limited resources, for example patients competing to book an appointment with an oncologist. In contrast, infinite server queues represent situations in which agents’ waiting times are mutually independent; for instance, the time it takes for one patient to develop cancer is not usually considered to be influenced by other patients. This independence makes infinite server queues simpler to analyze mathematically than their finite server counterparts. Finite server models are used to forecast short-term demand for beds in an intensive care unit in a hospital, where the focus is also on capacity planning [23]. A similar approach is used to optimize the number of beds in clinical wards with the goal of reducing the number of admissions turned away [24]. The authors study a queuing model with seasonal time-dependent arrival patterns and made approximations based on simpler infinite server queuing models. Staff in an emergency department are also a limited resource and as such the optimal allocation of their time is important for reducing patient wait times while minimizing costs. A queuing network model involving multiple patient types and time-varying demand is used to match peak staffing levels to peak forecast demand to meet hospital targets [25]. Similarly, a dynamic resource allocation algorithm based on a queuing network model is employed to improve patient length-of-stay in an emergency department by altering staffing in response to demand surges [26].

Infinite server queuing models [27] can be used as a more tractable approximation of finite server queues: lower bounds on congestion in finite and possibly saturating resource models are found by considering their infinite resource counterparts. Applications include modeling the number of inpatients on a ward [28] or in a network of hospital wards [29], traffic of patients in a hospital [30], and an emergency department with a view to quantifying the probability that patients must be diverted to another hospital [31]. A queuing network model and numerical study of colorectal cancer screening [32] is used to derive the capacity needed by a given system or a given population size to guarantee a certain service level in terms of patient waiting times to be screened. The model includes imperfect adherence to screening guidelines and analyzes both routine screening for average-risk patients and the additional resources required for surveillance of high-risk patients. Another multiserver, multiphase queuing network model and simulation study of cancer screening [33] is employed to identify optimal staffing levels and screening frequency in order to assess the impact on reducing the number and length of overdue screenings. A discrete time queuing model and simulation study investigates various interventions designed to reduce appointment and diagnostic delay in a hospital after the discovery of suspicious breast tissue [34].

The literature described above focuses on capacity planning such as optimizing the number of staff and hospital beds in the face of fluctuating demand. In contrast, we use queuing models for quantifying and comparing the benefits of medical interventions in terms of patient survival. Many of the papers described above use finite server queues as models of saturating resources, or infinite server queues as a more tractable but not ideal approximation. In contrast, we consider networks containing infinite server queues not as an approximation but as a phenomenological choice designed to be an exact model of the cancer screening applications. Infinite server queues act as a natural model for processes in which agents independently make state transitions in parallel. Specifically, the times it takes individual patients to develop a tumor are independent of each other. Our approach of exactly calculating performance measures to describe the outcomes of new screening technologies represents a novel application of queuing models in healthcare. Simulations are not necessary as we analyze the model exactly in the stationary regime, allowing us to quantify the benefits of screening and develop an associated optimal screening program. We first set out to develop our mathematical modeling platform, which we then apply to the example of pancreatic cancer data before deploying it more generally to data from different cancer types. R packages on the CRAN repository such as [35] can be used to numerically analyze queuing network models, which is particularly useful for large networks.

## Materials and methods

### Mathematical background

Our mathematical modeling framework is underpinned by the theory of queuing networks (S1 Appendix). Queuing theory is the formal, mathematical study of networks of waiting lines. The length of a queue is represented as a non-negative, integer-valued stochastic process. Formally a queue is described by detailing an arrival process, a service time distribution, and the number of servers operating at the head of the queue. This approach is succinctly summarized by Kendall’s X/Y/Z notation, where X specifies the arrival process, Y the service time distribution, and Z is the number of servers [36]. For instance, the M/G/∞ queue has Markovian arrivals (a Poisson point process), general (arbitrary) service times, and an infinite number of servers (meaning all customers are served in parallel). The equilibrium length of the M/G/∞ queue with arrival rate *λ* and mean service time 1/*μ* has a Poisson distribution with mean *λ*/*μ* [21]. Here we focus on infinite server queues, but in S1 Appendix we discuss examples that go beyond this paradigm.

A network of *J* queues is specified by describing the aforementioned aspects of each queue and the topology, which specifies the allowable state transitions, thereby detailing how customers are routed between queues. The latter is encoded by a *J*×*J* routing matrix *R*, whose *ij*^*th*^ entry, *r*_*ij*_, details the probability of being routed to queue *j* upon service completion at queue *i*. We use the convention that $r_{i0}=1-\sum_{j=1}^{J}r_{ij}$ is the probability of exiting the network upon leaving queue *i*. External arrivals to our networks are Markovian, such that new agents arrive according to the increments of independent homogeneous Poisson point processes with rates *η* = (*η*_1_,…,*η*_*J*_), but waiting time distributions between state transitions are arbitrary. In particular, the wait times are not assumed to be Markovian; we only assume that for a given queue *j* they are independent and identically distributed with finite mean 1/*μ*_*j*_. The total or aggregate arrival rates *λ* = (*λ*_1_,…,*λ*_*J*_) into each queue are the superposition of exogenous and internally rerouted arrivals. Formally, *λ* = *η*+*λR*, where all vectors are understood to be row vectors. This linear simultaneous system of equations is known as the traffic equations.

The stochastic process *N*(*t*) = (*N*_1_(*t*),…,*N*_*J*_(*t*)) describes the number of customers in each queue of the network over time and under the above assumptions is an instance of a type of network due to Baskett, Chandy, Muntz and Palacios (a BCMP network) and as such obeys the BCMP theorem [37]. The full joint stationary distribution of the number of customers in the network is given by the following input-output relation:

$$P(N_{1}^{*}=n_{1},\ldots ,N_{J}^{*}=n_{J})=\prod_{j=1}^{J}\frac{e^{-\rho_{j}}\rho_{j}^{n_{j}}}{j!}$$

when all queues have infinitely many servers, and where *ρ*_*j*_ = *λ*_*j*_/*μ*_*j*_ and superscript stars denote stationary quantities. In other words, at equilibrium, each queue in the network behaves as though it were an independent M/G/∞ queue whose length follows a Poisson distribution with mean *ρ*_*j*_.

One performance measure of a network is the average sojourn time, denoted by *E*(W*)–the expected time spent in the network at stationarity. This quantity can easily be computed using linearity of expectation and Little’s Law [38], which relates the average sojourn time to the average number of customers in the network, denoted *E*(N*), with the following exact input-output relation:

$$E(W^{*})=\frac{E(N^{*})}{\eta }=\frac{1}{\eta }\sum_{j=1}^{J}E\left(N_{j}^{*}\right)=\frac{1}{\eta }\sum_{j=1}^{J}\rho_{j},$$

where *η* is the long run average exogenous arrival rate into the network. See S1 Appendix for more details of queuing theory. Quantitatively solving the traffic equations and calculating performance measures of a network can be done using R packages such as [35], though the output they provide is numerical and not algebraic.

### The data

We apply our modeling framework to several different datasets from the Surveillance, Epidemiology and End Results (SEER) program [39], version 8.3.6. Using SEERStat, we obtain data from the years 2000 to 2016 including cancer of the pancreas, esophagus, kidney, liver, mesothelioma, and ovary. The database contains the age at diagnosis, survival and treatment type, patient age, ancestry and sex. Table 1 provides an overview of the data used.

**Table 1:** A summary of the SEER cancer incidence data used in the modeling and comparison of putative screening programs.

	Esophagus (N = 56445)	Kidney (N = 196685)	Liver (N = 91436)	Mesothelioma (N = 10092)	Ovary (N = 85530)	Pancreas (N = 147680)	Overall (N = 587868)
Sex							
Female	11303 (20.0%)	70726 (36.0%)	22093 (24.2%)	2017 (20.0%)	85530 (100%)	70213 (47.5%)	261882 (44.5%)
Male	45142 (80%)	125959 (64.0%)	69343 (75.8%)	8075 (80.0%)	0 (0%)	77467 (52.5%)	325986 (55.5%)
Age Category							
25–29	0 (0%)	592 (0.3%)	0 (0%)	0 (0%)	782 (0.9%)	0 (0%)	1374 (0.2%)
30–34	0 (0%)	1990 (1.0%)	0 (0%)	0 (0%)	1436 (1.7%)	201 (0.1%)	3627 (0.6%)
35–39	212 (0.4%)	4100 (2.1%)	316 (0.3%)	0 (0%)	2165 (2.5%)	713 (0.5%)	7506 (1.3%)
40–44	752 (1.3%)	8098 (4.1%)	1409 (1.5%)	0 (0%)	4429 (5.2%)	2406 (1.6%)	17094 (2.9%)
45–49	2314 (4.1%)	13549 (6.9%)	4900 (5.4%)	104 (1.0%)	7350 (8.6%)	5490 (3.7%)	33707 (5.7%)
50–54	4575 (8.1%)	20016 (10.2%)	10873 (11.9%)	383 (3.8%)	9967 (11.7%)	10214 (6.9%)	56028 (9.5%)
55–59	7097 (12.6%)	25790 (13.1%)	16476 (18.0%)	639 (6.3%)	11187 (13.1%)	15604 (10.6%)	76793 (13.1%)
60–64	8812 (15.6%)	28823 (14.7%)	16431 (18.0%)	1142 (11.3%)	11320 (13.2%)	20251 (13.7%)	86779 (14.8%)
65–69	9593 (17.0%)	29798 (15.2%)	13412 (14.7%)	1533 (15.2%)	10645 (12.4%)	23201 (15.7%)	88182 (15.0%)
70–74	8893 (15.8%)	26132 (13.3%)	11126 (12.2%)	1936 (19.2%)	9778 (11.4%)	24112 (16.3%)	81977 (13.9%)
75–79	8089 (14.3%)	22219 (11.3%)	9551 (10.4%)	2298 (22.8%)	8913 (10.4%)	24190 (16.4%)	75260 (12.8%)
80–84	6108 (10.8%)	15578 (7.9%)	6942 (7.6%)	2057 (20.4%)	7558 (8.8%)	21298 (14.4%)	59541 (10.1%)
Ancestry							
African American	6668 (11.8%)	22642 (11.5%)	12026 (13.2%)	0 (0%)	6885 (8.0%)	18091 (12.3%)	66312 (11.3%)
Asian/ Pacific Islander	1934 (3.4%)	9021 (4.6%)	14454 (15.8%)	0 (0%)	6183 (7.2%)	9716 (6.6%)	41308 (7.0%)
Caucasian	44149 (78.2%)	139920 (71.1%)	47688 (52.2%)	9414 (93.3%)	62426 (73.0%)	105221 (71.2%)	408818 (69.5%)
Hispanic	3694 (6.5%)	25102 (12.8%)	17268 (18.9%)	678 (6.7%)	10036 (11.7%)	14652 (9.9%)	71430 (12.2%)
Survival (months)							
Mean (SD)	25.3 (36.6)	77.3 (53.1)	22.5 (32.7)	16.1 (22.1)	56.6 (51.3)	13.5 (23.6)	43.7 (50.2)
Median [Min,Max]	10.0 [0,199]	75.1 [0,203]	8.13 [0,195]	9.00 [0,185]	43.8 [0,198]	5.00 [0,193]	20.0 [0,203]

We investigate six cancer types (esophagus, kidney, liver, mesothelioma, ovary, and pancreas) which currently do not have screening programs for the general population. We display a summary of the incidence data stratified by age, sex and ancestry and report summary statistics of survival. Absolute numbers appear on the left with percentages in parentheses.

### The mathematical modeling framework

**Fig 1:**
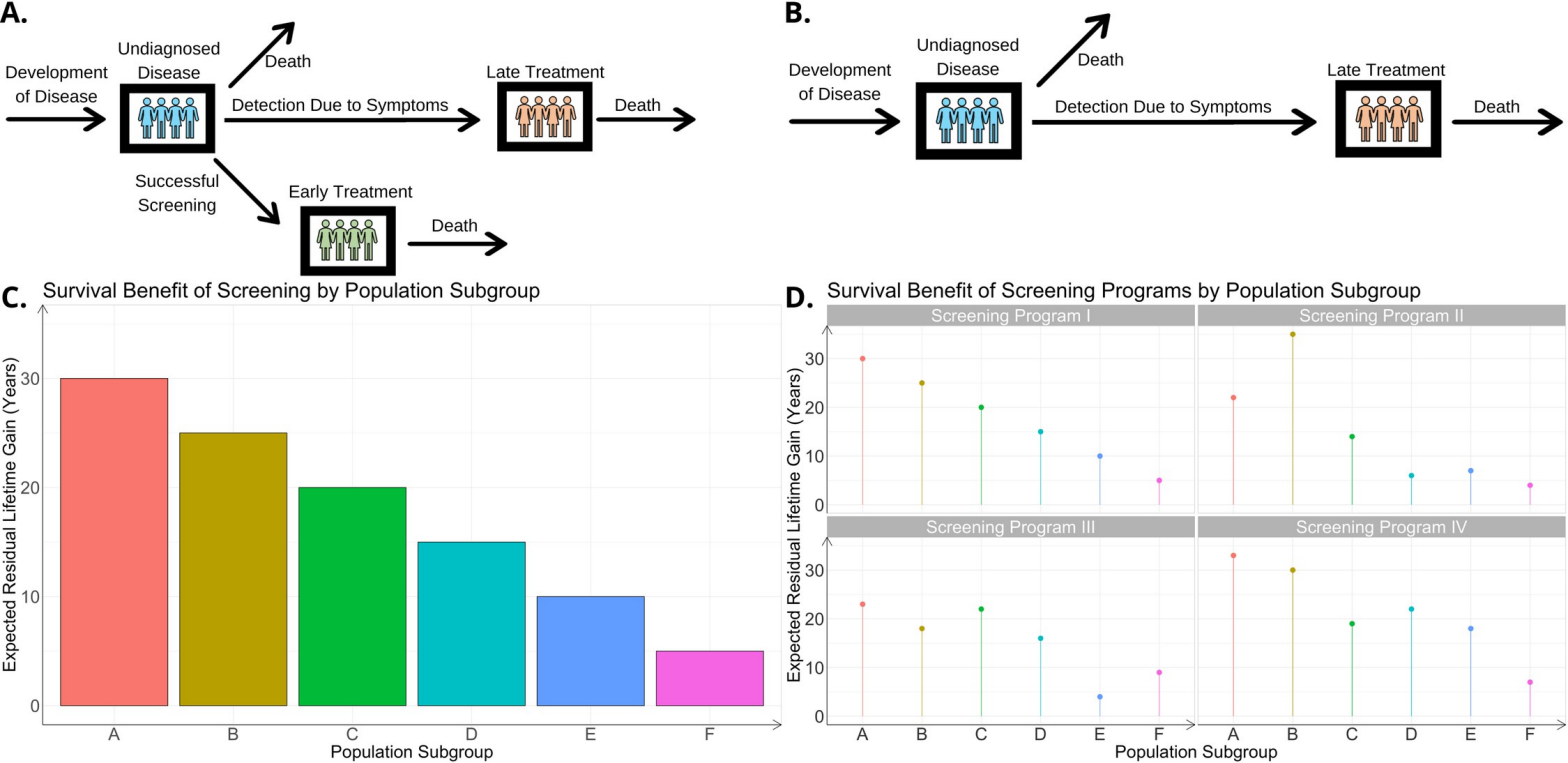
Outline of the modeling framework. (A) Consider a disease with poor survival which is typically detected late due to the onset of symptoms but for which early detection could improve survival. The number of individuals in each disease state is modeled by the occupancy of a queue. Analysis of the queuing network yields an estimate of overall survival. (B) Comparison of the results obtained from a network with and without screening allows quantification of the potential screening benefit. (C) Each population subgroup has its own identically structured model parameterized using available data stratified by relevant covariates such as age and sex. Population subgroups identified by relevant covariates are labeled A, B, C, etc. We then use these estimates to obtain numerical scores for an effectiveness metric of screening various groups, which suggests an optimal allocation strategy: rank subgroups by their scores and apply screening in the order of the ranking until exhaustion of screens or until screening is no longer considered cost effective for that survival benefit. (D) We then compare multiple different screening programs or potential screens of differing effectiveness levels, enumerated I, II, etc., for the various population subgroups A, B, C, etc., identified by covariates.

We design a mathematical modeling framework based on the theory of queuing networks (Fig 1, S1 Appendix, including code availability at https://github.com/evanhsph/Dean_et_al). We consider the scenario of a disease that becomes symptomatic at a late stage, at which point survival is short (Fig 1A), but for which early screening can result in significantly life-extending treatment (Fig 1B). We compare a model with different screening scenarios to one with no screening to ascertain the utility of screening. The model tracks the number of individuals with undetected early-stage disease, those with detected early-stage disease, and patients with late-stage disease. Late-stage disease corresponds to the patient data in SEER, which for diseases with no widespread screening (such as pancreatic cancer) are typically diagnosed at a symptomatic stage. Undetected early-stage disease represents the time from initiation of disease until detection (through screening or otherwise). Detected early-stage disease represents patients that are modeled to have been detected by a novel screen while pre-symptomatic. We do not use fine-grained information on clinical stages because SEER does not have consistently defined staging throughout, but this could be incorporated with cleaner data. For each disease state, there is a queue in the network representing the size of the population in that state (for instance early-stage disease). Waiting corresponds to the time that elapses before a state transition occurs. Patients enter the network and can move between different states of the network by being screened and found to have early-stage disease, by progressing from early- to late-stage disease, and by dying; these transition probabilities are described by a routing matrix.

Solving the traffic equations of the network allows specification of the stationary distribution for the number of customers in the network; basic performance analysis using Little’s Law yields the expected sojourn time in the network. This quantity can be interpreted as the expected residual lifetime, since exiting the network corresponds to death. We then compare this result to that obtained from an identical network with a different screening schedule to compare schedules, or to an altered network without screening to assess its effectiveness. The difference in expected residual lifetime in a network with screening and its counterpart without screening represents the expected residual lifetime gain. Once these closed-form expressions of improvement are found, we fit parameters using the data to obtain one parameter set for each relevant set of covariates; this approach yields numerical scores for the expected residual lifetime gain for each relevant subgroup of the population (Fig 1C). Note that the per cancer patient benefit is not the same as the per screen benefit. Since most patients screened do not have cancer, the benefit per screen is significantly lower than the benefit per cancer patient screened; a calculation of the per screen benefit incorporates an estimate of incidence. Our model tracks the number of cancer patients in various disease states so reported lifetime gains are measured per cancer patient.

### Parameter estimation

We utilize the SEER data for parameter estimation of our models. For the model without screening, we estimate the rate at which patients are diagnosed with cancer, the fraction that receive treatment before dying, the average time between diagnosis and treatment initiation, and the average time that patients receiving treatment survive. Note that the latter two quantities cannot be understood, in general, as rates, since the waiting time distributions modeled are much more general. For the model with screening, we additionally assign parameter values for the fraction of patients that are successfully screened and the average survival time for patients that are treated early because of screening. To assign parameters for the scenario involving screening, we transform the original data to generate synthetic data based on an assumption of the effectiveness of the intervention enabled by early detection. For instance, an early-detected cancer patient might have double the survival of a patient who did not opt for screening, with the factor 2 being a parameter in the model. This parameter can be changed and the effects of changes on model predictions investigated.

The fraction of patients that receive treatment before dying is calculated by averaging over the empirical fraction of patients in the SEER database that receive treatment each year from 2000 to 2016. The average time that patients receiving treatment survive is calculated by averaging over the difference between the date of death and the date of treatment initiation of all patients who receive treatment. The rate per year at which patients are diagnosed with cancer is determined by the average incidence data by year, where we average over the numbers in each year from 2000 up to 2016. To estimate the number of patients that develop cancer (initially undiagnosed) we use the incidence average as an approximation, which might suffer from a (small) underestimation stemming from those patients that died, whether from cancer or another cause, before diagnosis. The older the population subgroup, the worse this approximation becomes as more people die of competing risks. This effect could be corrected for by estimating the number of deaths from competing causes between tumor initiation and diagnosis for each population subgroup; however, in all but the very oldest age groups this effect is small and does not change the relative ordering of overall survival estimates. The average time that patients receiving treatment survive is estimated by the average survival time from the SEER data. This approach is affected by the right-censoring of the data, in that many patients from the dataset are still alive. For these patients we impute survival times using conditional empirical survival distributions, i.e., using data on patients who have died, we calculate how long they typically survive, given that they had survived a certain amount of time. We make a similar adjustment when modeling screening and the survival benefits it confers. If a patient has cancer detected early as a result of screening and then lives to their residual life expectancy (or a certain fraction thereof), then this modeled residual survival time stems from their conditional life expectancy given they have lived to their current age. The data for the conditional life expectancies are extracted from [40],[41] and are stratified into patient groups up to age 64, 65-74-year-olds, and people aged 75 and up for each ancestry and sex combination. This discrete stratification results in a slight artificial upward bump in estimated survival times at age 65. Discretizing in this way means that our results do not quite match the monotonically decreasing trend that one expects. With more fine-grained data on conditional life expectancies, one could avoid such artifacts.

Another potential censoring issue arises from the fact that separate models track the number of individuals of each age group and that individuals’ age category changes over time. Compartmental and Markov chain models face the same issue. To adjust for this effect, separate models do not track the number of patients of a certain age, but the number who developed disease at a certain age. Thus, there is no flux of individuals between models or age categories over time.

An alternative approach is to reinterpret exiting the network as either dying or aging out of that age category; this implies that customers entering the network represent new cancer patients or existing patients entering that age category. Another alternative is to route customers between a succession of networks as agents change age category. If the typical sojourn time in the network is significantly shorter than the age range covered (as can be the case with cancers with poor survival), then these adjustments make little difference. Otherwise, we can coarsen the age groups to cover a longer period. As with other mathematical models of screening, it is often necessary to estimate when people first develop cancer, or when they have cancer that is detectable by screening. An example in the setting of pancreatic cancer is given by the modeling in [42]. Because our model calculates overall survival estimates based on adjusted life expectancies, these estimates are not confounded by lead time bias.

We do not have reliable information on the average time between diagnosis and the start of treatment, and this quantity can vary substantially between different institutions and geographic locations. This value is therefore represented by a tunable parameter in the model. Because the queuing model makes no assumptions about the parametric form of waiting time distributions or distributions governing routing probabilities, we cannot use standard methods such as maximum likelihood estimation or method of moments because there is no assumed likelihood or particular distribution whose moments are known. Averages are calculated from empirical means and routing probabilities from empirical proportions. This level of simplicity and transparency is a welcome feature of this modeling approach.

### An optimal allocation algorithm

Given the modeling framework and parameter estimation method outlined above, we then utilize the patient data described in the Data section to estimate parameters of the models and quantify the benefits of screening. Since information is available on patient characteristics such as sex, ancestry, and age, we estimate a separate parameter set specific for each population subgroup. This approach leads us to design an optimal allocation algorithm consisting of the following steps: 1. Set a minimal lifetime gain T above which the screen is considered worthwhile. 2. Consider the subgroup with the highest modeled lifetime gain. If this exceeds T, then allocate screens to this group. 3. If there are more screens available than members of this first subgroup, then consider the second ranked group. If their lifetime gain exceeds T, then allocate screens to them. 4. Repeat this process of saturating the next highest ranked subgroups until exhaustion or until the survival benefit from screening falls below T. Extensions of this algorithm such as repeated screens are discussed in S1 Appendix and depend on the level of dependence between test outcomes on an individual patient. This approach can be used to compare the presence or absence of a screening schedule as well as to compare various screening schedules against each other. Fig 1D shows a comparison of four different hypothetical screening schedules, for instance represented by potential screens with differing detection probabilities and resultant survival benefits in arbitrary population subgroups. The algorithm described above aims to maximize the number of life years saved as a representative example of our approach, but there are other potential considerations when designing a screening program; for instance, one may choose to spread screens evenly across different population subgroups with different ancestries. Survival estimates here are based on observational data, which may be confounded by socio-economic status, access to care, quality of care etc. Survival estimates may be improved if randomized controlled trial data was available.

### Treatment-associated morbidity and mortality and test specificity

Morbidity, mortality, and test specificity can naturally be incorporated into our modeling framework. Morbidity associated with cancer treatment [4] is modeled by multiplying survival times (i.e., sojourn times) by a QALY factor between zero and one, thus reweighting the survival period by its quality. A multiplicative factor of one represents no reduction in quality of life, while smaller factors represent treatment-associated morbidity. If side effects are just temporary, then the period when they occur is reweighted by a factor less than one, but the period afterwards is not. As an example, Fig B in S1 Appendix shows quality adjusted survival for patients treated early for pancreatic cancer. As morbidity increases, the QALY factor drops, resulting in reduced quality-adjusted survival due to early screening. Treatment-associated morbidity is likely to depend on the cancer stage at detection, which can be considered in our approach.

Treatment-associated mortality is incorporated by adjusting the service time distribution corresponding to the treated population. If *p* is the probability of treatment-induced mortality, then with probability *p* the service time is very short, representing premature death, and with probability 1−*p* it is the original service time distribution. The probability *p* and the reduced service time distribution (a point mass at one month) are both tunable parameters. The reason for the latter placeholder is that we do not have information on treatment-associated mortality when patients are diagnosed earlier due to a hypothetical screen. However, the exact distribution selected has far less influence on the average survival than the probability p. Fig C in S1 Appendix displays the average survival gain from early screening for pancreatic cancer for varying mortality and treatment effectiveness. The higher the risk of mortality, the lower the average survival gains become when holding treatment effectiveness constant. Treatment-associated mortality likely depends on the cancer stage at diagnosis; while the mortality risk of pancreatic resection may be high, early detection may result in less risky surgery or less aggressive chemotherapy than the risk associated with aggressive treatment such as a pancreatectomy [43].

As the specificity of a screen decreases, there are more false positives which can result in financial or psychological costs and treatment-related morbidity and mortality. To incorporate this feature into our methodology, we modify the network topology and the routing probabilities. We split the early treatment queue into separate queues for false positives and true positives which have different service time distributions. In practice a confirmatory test or scan is used following a positive screen result before commencing any treatment, which corresponds to adding another queue that is routed to from queues representing positive screen results, and from which negative scans would be routed or rerouted to a queue representing the healthy population. Since cancer is rare, even a small rate of false positives could lead to many healthy people requiring confirmatory scans. A fraction of those might still be false positives and unnecessarily treated and therefore, population level screening can be problematic, and a more targeted approach based on risk factors such as family history or chronic disease may be preferred [44]. If test errors are uncorrelated or weakly correlated, then rescreening may eliminate many false positives.

Fig D in S1 Appendix considers a pancreatic screening program under various false positive rates and levels of treatment effectiveness. These rates are a tunable parameter of the model but are low in the example used because we suppose that the confirmatory scan is very specific. Census data is used to estimate the number of healthy individuals in this subgroup [45] and SEER data [39] for the number of pancreatic cancer patients. The false positive individuals experience reduced survival times by a tunable parameter. Lowering specificity reduces the net benefits of screening due to more overtreatment. This net score is negative when early screening causes overtreatment of healthy individuals to outweigh the benefits of earlier detection.

## Results

### Assessing the benefits of pancreatic cancer screening

Pancreatic ductal adenocarcinoma is a particularly deadly form of cancer with limited treatment options and low overall survival [46],[47],[2]. By the time it is detected, it has often progressed to metastatic disease with poor prognosis [48], [49]. Currently no widespread pancreatic cancer screen is available, but several approaches are under investigation, for instance a cell free DNA-based screen for early diagnosis [50]. With early detection, pancreatic cancer patients may receive potentially curative treatment [51], and evidence from genomic sequencing indicates a 15-year period of genetic progression from disease initiation to the metastatic stage, suggesting a sizable window during which screening would be beneficial [52]. Screening for PDAC is not standard amongst the general population because of its low incidence and lack of a highly sensitive and specific test [53]. However, high-risk individuals, i.e. those with a family history, genetic predisposition or chronic disease, generally have access to screening modalities [44]. Previous approaches investigate the effectiveness of endoscopic screening of high-risk individuals [54] and the potential benefits of biannual MRI scans [55].

We first model the potential utility of a novel pancreatic cancer screen under different scenarios of its ability to detect early-stage cancer and the resultant survival benefits per cancer patient. To this end, we compare the results of a queuing network with screening to one without. Exact analysis of these queuing networks yields qualitative descriptions of the screening benefits in terms of model parameters for each population subgroup. This analysis is performed as outlined in detail in sections 2.3 and 2.4 in S1 Appendix, where equation (2) displays exact distributional results for the number of individuals in each state and equations (3), (4), and (5) show exact input-output relations between model parameters and quantities of interest. We first use the network without screening to estimate parameters from the SEER pancreatic cancer epidemiological data. Since we cannot estimate the effectiveness of a hypothetical screening strategy directly from the data, we investigate this quantity by adjusting model parameters as follows. For our purposes there are two most relevant axes along which a screening program can be assessed; its ability to detect early-stage cancer and the survival benefit conferred given that a cancer is successfully detected by screening at an early stage. The latter we define to be ‘effectiveness’ and measure it on a percentage scale representing how much life expectancy is added compared to a healthy individual of that group. For ease of discussion, we here set specificity to 100%, although this is a parameter of the model that can be adjusted. We consider several different screening scenarios by varying the detection probability and effectiveness.

In our model, we stratify the population into subgroups according to clinically relevant covariates to the extent that they are available in the epidemiological data. If a subgroup falls below 100 subjects, then we exclude it from the analysis: for instance, Native Alaskans and people under 25. The reason for choosing this level of granularity is that grouping too coarsely may mask potentially relevant differences within a subgroup, whereas grouping too finely yields smaller sample sizes and therefore less reliable statistical parameter estimates.

**Fig 2:**
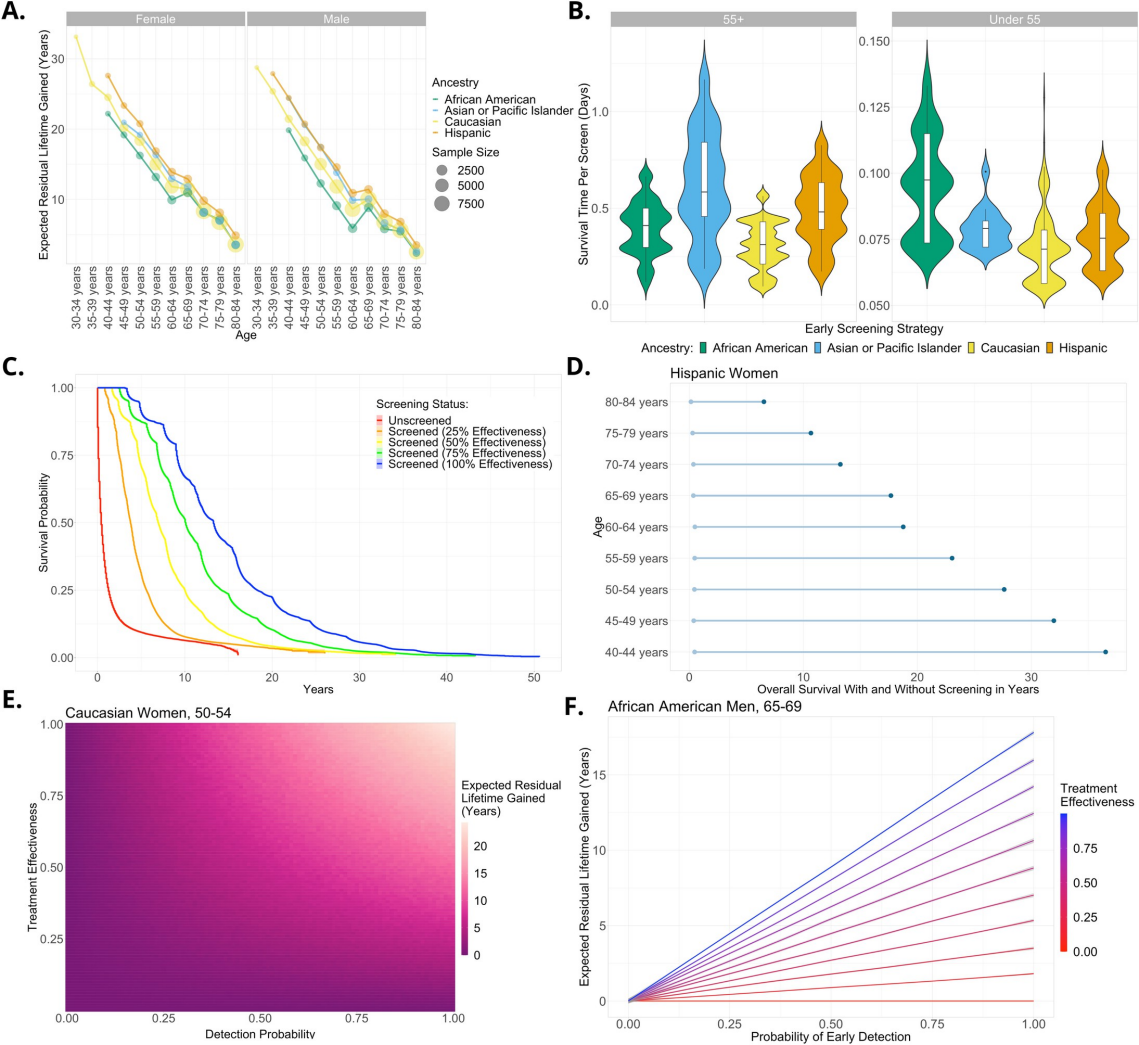
Survival benefits of screening for pancreatic cancer under a range of scenarios. (A) Overall survival benefit conferred from an example scenario of pancreatic cancer screening stratified by sex, ancestry, and age assuming that early screening never yields false negatives and identifies cancer early enough to achieve 75% effectiveness (survival to 75% of the average life expectancy for that group on average). The slight upward bump at age 65 is due to the conditional survival distribution given having lived to a certain age and its discrete stratification (Materials and Methods). (B) Under the assumptions of panel A we compare various screening programs targeted at different population subgroups based on age and ancestry. The survival benefit per pancreatic cancer screen (i.e., incidence adjusted) is shown. (C) Survival curves comparing overall survival from screens with different levels of effectiveness and the unscreened. (D) Difference in overall survival with (right dot) and without (left dot) screening for Hispanic women under assumptions of panel A. (E) Overall survival benefit for Caucasian women, aged 50–54 as a function of screen detection probability and resultant treatment effectiveness. The color of the heatmap represents the expected residual lifetime gain and is calculated exactly from the model. (F) Overall survival benefit to African American men, aged 65–69 as a function of model parameters: detection probability and resultant treatment effectiveness. Each contour represents a different scenario of benefit of early detection. Increasing the screen sensitivity and resultant treatment effectiveness can drastically change predicted overall survival.

We first investigate the scenario in which screening effectiveness is 75%. When using the SEER pancreatic cancer data for estimating the screening benefits, we find that younger patients experience the largest benefit per positive case detected (Fig 2A). This finding is expected since younger patients live longer upon receiving treatment than older patients, and the model provides an exact quantification of this relationship. For example, 30–34-year-old Caucasian females can expect to live over 30 more years on average (first and third quartiles 28.5 and 31.5 respectively), whereas their 50–54-year-old counterparts on average gain about 20 life years (IQR of 33 months) under these modeling assumptions. We find that 60-64-year-old Hispanic males can expect to live about 11 years extra on average, whereas their Caucasian counterparts are predicted to live for an additional 9 years on average (both have IQR of about 3 years). This observation reflects the modeling assumption that individuals live to a percentage of their respective life expectancies, which differ by group. We find that age is the dominant covariate (Pearson correlation -0.6 with modeled survival) when determining survival. The framework can be used to investigate alternative scenarios, for instance those in which there is differential effectiveness or uptake depending on ancestry, sex, or age.

We next compare several different screening programs with a constraint on the total number of screens available (Fig 2B). For instance, for a given level of effectiveness (e.g., 75%) we investigate the effects of allocating a fixed number of screens across different population subgroups and display the survival benefit achieved per screen (not per cancer patient). Data on differential incidence is obtained from [45]. When investigating the differential impact of screening for pancreatic cancer, we find that the largest benefit is achieved by screening older patients (those over 55) since their incidence is significantly higher, and therefore fewer screens are needed to identify each positive case. For instance, on average, screening Asian and Pacific Islanders aged 55 and over confers 0.6 days of extra life per screen, whereas screening their younger counterparts achieves just under 0.1 days extra per screen. This age relationship based on differential incidence is found across ancestries. The findings of this comparison suggest that targeting subsections of the population can vastly increase the benefit of a screening program. Our results imply that screening Asian and Pacific Islanders over 55 would be most efficacious. However, if there are more screens available than the size of that group, then our results suggest that other over 55-year-olds of different ancestries would be the next most efficacious groups to screen (Fig 2B).

We then investigate the survival benefit of screening depending on the screening effectiveness (Fig 2C). When averaging over all population subgroups (Fig 2A), we find that, as expected, a more effective screen increases the survival benefit linearly, and our model allows us to quantify this relationship. For instance, the 10-year survival probability of unscreened individuals is approximately 7%, whereas under the assumption of 100% screening effectiveness it is around 65%. When investigating specific population subgroups, we again observe that more effective screens lead to a bigger survival benefit, but the quantitative estimate of the benefit depends on sex, age and ancestry. The predicted survival of female Hispanic pancreatic cancer patients of different ages with and without screening is shown in Fig 2D while all other groups are displayed in Fig E-J in S1 Appendix. The left point of the dumbbell represents survival without screening and the right point is with a screen of 75% effectiveness. In general, we find that younger patients enjoy a larger benefit per positive case detected from screening than older patients. Based on these results, we predict for instance that 40–44-year-old Hispanic women on average gain around 36 life years per positive case detected, compared to less than 15 years for their 70–74-year-old counterparts (Fig 2D).

An advantage of the queuing network approach is the ability to exactly analyze the relationship between inputs and outputs (see equations (3)-(5) in S1 Appendix). This ability allows us to investigate how changing one input model parameter influences the results while holding all other parameters fixed. The results for 50-54-year-old Caucasian women and 65-69-year-old African American men are shown in Fig 2E and 2F as representative examples. The heatmap in Fig 2E shows the exact modeled average survival gain per patient detected from screening calculated analytically from the model. We find that increasing the sensitivity of the screen from 50% to 100% increases survival by about 10 years under the most effective scenario (100% effectiveness). In Fig 2F, each contour represents a different screening effectiveness as the detection probability is varied. We find that changing the effectiveness or sensitivity of the screen can drastically alter predicted overall survival. For instance, when the probability of detection is 100%, a change of effectiveness from 50% to 100% changes the expected residual lifetime gain from about 9 to about 18 years.

### Testing distributional predictions of the BCMP theorem

**Fig 3:**
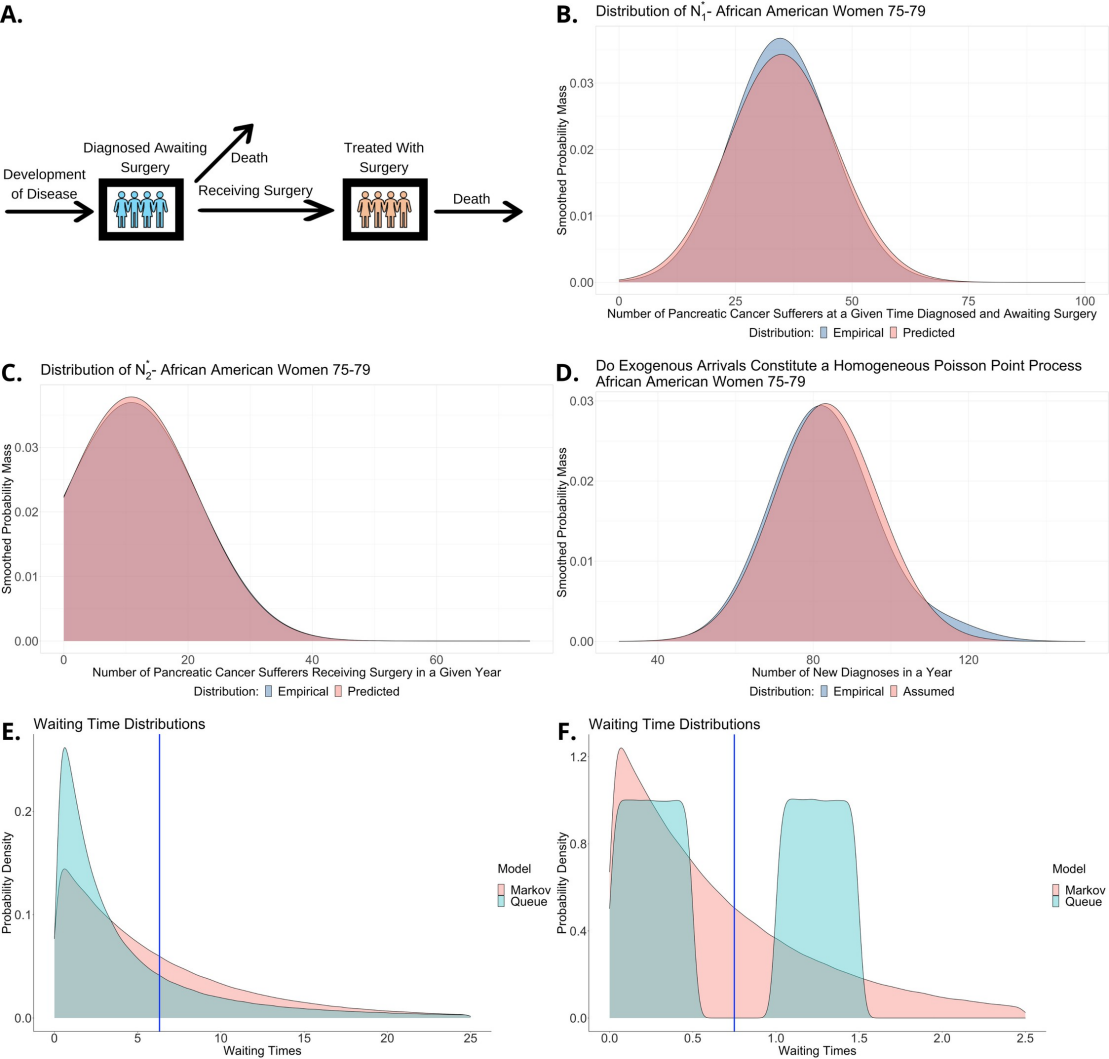
Distributional descriptions of the modeling framework. (A) Schematic queuing model of pancreatic cancer diagnosis and surgery. (B) Testing the distributional predictions of the BCMP theorem for the number of diagnosed pancreatic cancer cases among African American women aged 75–79 awaiting surgery as a particular example. We compare the predicted distribution of $N_{1}^{*}$ from the model and the empirical distribution of this quantity from the incidence data. Probability mass functions are kernel smoothed to ease visualization. (C) Testing distributional model predictions for the number of African American women aged 75–79 with pancreatic cancer each year who receive surgery, $N_{2}^{*}$, against the empirical distribution from the data. (D) Testing whether pancreatic cancer diagnoses of African American women aged 75–79 follow the Markovian assumption for exogenous arrivals required by the BCMP theorem. (E) A model comparison of how waiting time distributions are approximated. A deterministic model uses the average only, Markov models approximate by the best fitting exponential distribution, whereas simulations and queuing models can use the ‘true’ distribution–in this case a lognormal distribution. (F) Comparing how a mixture waiting time distribution is modeled.

One advantage of the queuing-theoretic approach is the distributional description we can obtain (see equation (2) in S1 Appendix), which is useful when considering aspects of performance that go beyond averages, such as peak-load planning under conditions of stochastic demand. We would like to know, for example, not just how many early diagnoses would be made on average with a novel pancreatic cancer screen, but also the variability around this estimate. The BCMP theorem is the tool that allows for distributional predictions, but it applies only when certain assumptions are met [37]. To test the predictions of the theorem we consider the example of the number of pancreatic cancer diagnoses and resulting surgeries for patients. We cannot use the screening model from above to test predictions because we do not have data from this hypothetical screening program. Instead, we use an example where we do have data so that we can validate distributional results. Fig 3A shows a schematic representation of the model used to find predicted distributions.

Let *N*_1_(*t*) represent the number of individuals diagnosed with pancreatic cancer that have not received surgery as part of their treatment up to time *t* and *N*_2_(*t*) the number that have. We think of *N*_1_(*t*) and *N*_2_(*t*) as stochastic processes tracking the length of two infinite server queues. New diagnoses arrive according to a Poisson process of rate *η*_1_. Individuals with the disease (not treated with surgery) either die or receive surgery after random times whose means are 1/*μ*_10_ and 1/*μ*_12_ respectively. Hence, the random time until exit of an individual from the first queue is given by 1/*μ*_1_ ≔ 1/(*μ*_10_+*μ*_12_). A fraction *r*_10_ die and *r*_12_ receive surgery, hence *r*_10_+*r*_12_ = 1, and we set $r_{10}=\frac{\mu_{10}}{\mu_{10}+\mu_{12}}$ and $r_{12}=\frac{\mu_{12}}{\mu_{10}+\mu_{12}}$. We solve the traffic equations, parameterized in this case by:

$$R=\left(\begin{array}{cc} 0 & r_{12}\\ 0 & 0 \end{array}\right)\;\mathrm{and}\;\eta =\left(\begin{array}{c} \eta_{1}\\ 0 \end{array}\right).\;\mathrm{Hence},\;\lambda =\left(\begin{array}{c} \lambda_{1}\\ \lambda_{2} \end{array}\right)=(\begin{array}{c} \eta_{1}\\ r_{12}\eta_{1} \end{array}).$$.

By the BCMP theorem, this relationship in turn means that $N_{1}^{*}∼Poisson(\frac{\eta_{1}}{\mu_{1}})$ and $N_{2}^{*}∼Poisson(\frac{r_{12}\eta_{1}}{\mu_{2}})$. These are the predicted theoretical distributions for the number of individuals in each state. Note that we are not assuming exponential waiting times, so the stochastic processes *N*_1_(*t*) and *N*_2_(*t*) are not Markov chains and thus these distributional results cannot be derived with Markov chain methods under the same assumptions. For each population subgroup we estimate the parameters as detailed in the Materials and Methods section. We then compare the predicted distributions from the queuing model (one for each subgroup for each queue) to the empirically observed distributions from the SEER data. The predicted distributions are calculated solely based on incidence averages of the data from 2000–2016, and not on any other aspects of the data itself (such as raw counts which are the basis of empirical distributions). Fig 3B and 3C show the predicted and empirical distributions for $N_{1}^{*}$ and $N_{2}^{*}$ for one example population subgroup, African American women aged 75–79; all others are shown in Fig K-N and O-R in S1 Appendix for the first and second queue, respectively. The predicted and empirical distributions are both discrete, but we smooth out the probability mass functions for ease of visualization.

We additionally formally test whether the empirical and predicted distributions can be distinguished using the Kolmogorov-Smirnov test at the Bonferroni adjusted 5% level. We find that in only one case out of 76 population subgroups (Caucasian females aged 65–69) are there statistically significant differences to reject the null hypothesis that both distributions are the same. This corresponds to when the assumptions of Markovian exogenous arrivals of the BCMP theorem are a less good fit. Even in other cases where the predicted and empirical distribution are not statistically significantly different, the goodness of fit tends to correspond to how well the arrival data match the Markovian assumption (Table A in S1 Appendix). To assess this assumption formally, we test with the K-S test at the adjusted 5% level whether the number of exogenous arrivals for a period of fixed length are Poisson distributed as would be the case for a Poisson point process. To do so we set a time interval of length one year and then check whether the number of arrival increments during that period across different years follow a Poisson distribution with a mean given by the estimated arrival rate multiplied by the time period. This is necessary (but not sufficient) to be a Poisson process, so if this is violated then certainly the assumption of Markovian arrivals is too. Fig 3D shows an example of this comparison for African American women aged 75–79. Results for all other population subgroups are in Fig S-V in S1 Appendix.

### The benefits of queuing network models and a model comparison

There are several advantages of queuing models over their simulation, ODE and Markov chain counterparts. Table 2 shows a summary of differences between these modeling approaches. Note, the table details what the methods can do, not how they are used in every instance. For example, there are exact stochastic simulation models, but also some which are (justifiably) deterministic and approximate. Similarly, although some ODEs, Markov chains, and queuing network models are too complicated to analyze exactly (and in these instances are presumably not intended to be), they often can be.

**Table 2:** Summary Comparison of Modeling Techniques.

	Modeling Technique			
Property	Simulation	ODE	Markov Chain	Queuing Network
Discrete	Yes	No	Yes	Yes
Stochastic	Yes	No	Yes	Yes
Wait Times	Arbitrary	Deterministic	Exponential	Arbitrary
Distributions	No	No	Yes	Yes
Exact Formulae	No	Yes	Yes	Yes

A comparison of the advantages and disadvantages of simulation, ODE, Markov chain and queuing network models.

The number of customers in a queue is discrete, whereas a compartmental ODE model assumes all quantities are continuous, which is a poor approximation when numbers are small. For example, in a discrete model there may be one or two patients waiting to see the oncologist, but in a continuous model the effect of a non-integer number of patients waiting is included. The difference in modeling 1000 and 1000.36 patients is trivial, but the difference between 1 and 1.36 may not be. Whether this is an issue for our setting depends on the size of the population subgroup considered and the incidence of the specific cancer type. Similarly, the deterministic nature of ODEs is not an issue when considering aggregate measures of large populations. However, stochastic models can be useful for small populations (such as the number of patients with a rare disease waiting to see an oncologist) or when investigating metrics involving aspects of a distribution other than the mean, such as those relevant to resource allocation (staffing levels, number of hospital beds etc.), which depend not only on averages but on fluctuations.

Closed form expressions for distributions allow us to calculate exact probabilities of events of interest, including those more extreme than any observation seen so far–something that is difficult to approach with purely statistical methods. For instance, what is the probability of seeing twice the number of pancreatic cancer diagnoses in a year as has been seen before in the 20 years of collecting data? Whether this probability is 10^−2^ or 10^−6^ matters when planning for fluctuating demand and tradeoffs regarding the cost of redundant resources. Estimating such probabilities can be impractical with simulation models as each replicate must be run for a long time to observe such extreme values, which can be computationally expensive for complicated models with large agent populations. Using the model from Fig 3A as an example, we observe that the distributional results immediately yield the exact tail probability in terms of the model parameters:

$$P(N_{1}^{*}>x)=e^{-\frac{\eta_{1}}{\mu_{1}}}\sum_{k=\lfloor x+1\rfloor }^{\infty }\frac{{\left(\frac{\eta_{1}}{\mu_{1}}\right)}^{k}}{k!}=1-e^{-\frac{\eta_{1}}{\mu_{1}}}\sum_{k=0}^{\left\lfloor x\right\rfloor }\frac{{\left(\frac{\eta_{1}}{\mu_{1}}\right)}^{k}}{k!},\;for\;any\;x\geq 0.$$

It is tempting to calculate the steady state abundances of the compartmental model and claim that a reasonable stochastic approximation is that each steady state quantity follows a Poisson distribution with the calculated means (essentially justified by a Binomial approximation to the number of individuals in each state, with fixed probabilities of transit to each subsequent state, and then approximating this Binomial distribution by a Poisson distribution with matching mean). However, this approximation is invalid when there is a finite server queue in the network, such as patients waiting to see an oncologist or use an MRI machine which are more appropriately modeled by finite server queues (section 2.5 in S1 Appendix). The queuing approach of applying the BCMP theorem still works in this more general setting.

A stochastic model that extends the compartmental model is a Markov chain model. The rates of the ODEs become parameters of exponential waiting times. Queuing models are more general than Markov models, relaxing distributional assumptions on waiting times. These models are not restricted to constant hazard rates, but can use arbitrary waiting time distributions, provided they are supported on the positive real numbers and have finite mean. When fitting parameters, we do not have to know (potentially complicated) hazard rates of certain events, but simply their mean waiting times, i.e., we can use the empirical wait time distribution directly rather than a parametric model that requires fitting. In the Markov chain case, rates and the reciprocal of means coincide. But in general, this need not be true. This generalization includes more complicated models (such as those that vary with time) that Markov chains cannot handle (in that distributional results are lost under these more flexible assumptions). For instance, the hazard rate until individuals die typically increases over time. A memoryless exponential waiting time with a fixed rate is evidently a poor model for such a phenomenon.

Fig 3E and 3F show examples of waiting time distributions that are poorly approximated by the Markovian assumption. The queuing model uses the ’true’ distribution which in this case is log-normal and a mixture of uniform distributions, respectively. The Markov model fits the closest exponential distribution, with maximum likelihood suggesting estimating the rate by the reciprocal of the mean of the ’true’ waiting time distribution. The blue line shows the average wait time, which is all a deterministic model can capture of the ’true’ wait time distribution. The distribution in Fig 3F is pathological by design but shows that some distributions can result in a deterministic model that assumes wait times are a certain average value that occurs with probability zero. Even the best fitting Markovian distribution can be substantially inaccurate. For example, the log-normal distribution of Fig 3E with mean and standard deviation on the log scale of 1 and 1.3 respectively, has variance equal to 183.75, whereas the best fitting exponential distribution has variance of just 40.47. Similarly, the distribution in Fig 3F assigns zero probability to the service time being between ½ and 1, but the best fitting exponential assigns probability 0.135 to this possibility.

For complicated mathematical reasons (see [56]) the stationary distribution of the BCMP queuing network often coincides with that of the equivalent Markov chain. Here equivalent means replacing all service time distributions by exponential distributions with the same mean. This property of certain queuing models is called the Insensitivity Property, so named because the stationary distribution is insensitive to finer details of the service time distribution. So, an equivalent Markov model often fortuitously leads to the same distributional results. However, there are queuing network models for which this is not the case, though a discussion of sufficient conditions to possess this property is beyond the scope of this article (see [57]).

In fact, if one simply wishes to calculate stationary averages then numerical scores resulting from compartmental models, Markov chains, simulations, and queues all ought to coincide—compare equations (1) and (2) in S1 Appendix for example. This means that certain figures, such as Fig 2A would look identical under many different model types. We do not display identical plots from different models, but present a more nuanced discussion of modeling differences. The reason that we show some plots that could equally be generated using other techniques is twofold: firstly, some stationary averages are integral to the application–as decisions related to screening depend upon large populations, it is only natural that one compares aggregate metrics. Secondly, that the aggregate measures can be easily computed using the same queuing framework is convenient. We need not resort to another model or make more stringent assumptions to obtain these results. A priori it is not always clear how robust certain results are to assumptions, so obtaining identical results in a more general setting is informative.

Some outputs of these models are not commensurable—for example, distributional characterizations possible with exactly solvable queuing networks and Markov chains cannot be compared with ODEs which yield no such descriptions. Similarly, simulations may yield empirical distributions based on performing many replicates, but these do not offer the same qualitative descriptions as distributions with exact formulae for their parameters. The same is true of exact input-output relations of the queuing model that result from operational laws, such as those in equations (3)-(5) and (7)-(10) in S1 Appendix; simulations may produce the same quantitative answers but cannot provide the qualitative formulae that give a deeper understanding. This is an important difference but not one that is easily visualized. The other main difference of these modeling approaches (again not readily amenable to comparisons in plots) is the process required to use them. Analyzing the queuing network involves simple pen and paper calculations, whereas agent-based simulation models require writing code and (potentially) lots of compute time.

On our GitHub page we offer a simple example of a stochastic simulation of a two-compartment model. All wait times are exponential, so this is a simulated Markov chain. The approximate run times of the simulation for different agent populations are shown in Table 3. The run time depends on the efficiency of the code and the machine it runs on (using our code one can test the compute time on their machine), so the values in this table should be thought of as an illustration rather than a fixed benchmark. The compute time is linear in the number of agents, so simulating a large population (such as screening all 40-50-year-olds for cancer) can be time consuming. Moreover, using larger and more complicated networks increases run times further. The pen and paper approaches are solved in the abstract and so do not depend on population sizes (until parameter estimation, which is the same across all approaches).

**Table 3:** Scaling of Compute Time with Size.

	Compute Time			
Simulation Size	Simulation	ODE	Markov Chain	Queuing Network
**10**^**5**^ Agents	12 seconds	Instantaneous	Instantaneous	Instantaneous
**10**^**6**^ Agents	2 minutes	Instantaneous	Instantaneous	Instantaneous
**10**^**7**^ Agents	20 minutes	Instantaneous	Instantaneous	Instantaneous
**10**^**8**^ Agents	3.3 hours	Instantaneous	Instantaneous	Instantaneous

A comparison of time taken to calculate model outputs for populations of different sizes.

### Investigation of screening for different cancer types

**Fig 4:**
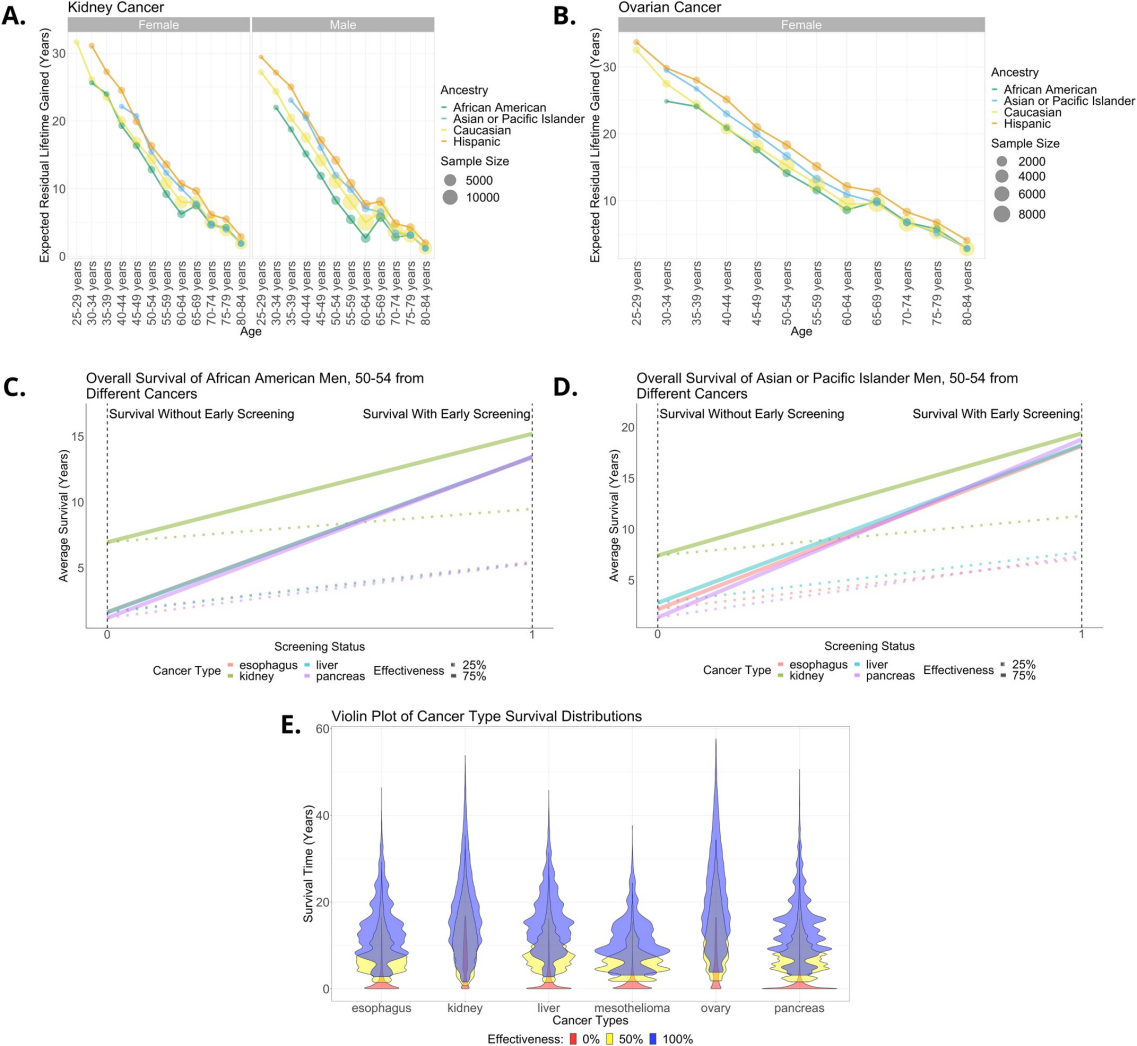
Overall predicted survival benefits of putative screening programs for a variety of cancer types for which there are currently no widespread screening technologies and a cross cancer comparison. (A) Overall predicted survival benefit from a potential kidney cancer screen of 75% effectiveness stratified by age, sex and ancestry. There is an artificial bump at age 65 due to the discrete stratification of conditional lifetime distributions (Materials and Methods). (B) Analogous plot for a potential ovarian cancer screen under the same assumptions. The sample size used to estimate the parameters is given by the point size. Higher sample sizes yield more reliable estimates, so where the sample size is less than 100 subjects, we do not make an estimate. Since this disease only affects females, males are not included. (C) Comparison of the change in overall survival as a result of screening for different cancer types for African American men aged 50–54. Survival without screening is shown on the left-hand side and the predicted survival with screening is on the right. We display two scenarios: one in which screening has an effectiveness of 25% and another with effectiveness of 75%. (D) The analogous slope chart for Asian or Pacific Islander men of the same age. (E) Violin plot showing the survival time distribution of cancer patients by type under three different scenarios. The first (in red) is based on the SEER data and is the observed survival distribution. Then we present two modeled screening scenarios: one in which effectiveness post detection is 50% (yellow) and another where it is 100% (blue).

We apply our modeling methodology to five other cancer types for which there are no widespread screening programs for the general population. Using SEER epidemiological data for these cancer types, we investigate the potential benefits of novel putative screening programs (Fig 4 and Fig W-X in S1 Appendix). These curves are determined as in Fig 2A. A summary of the SEER incidence data used in the modeling is provided by Table 1. Note that the data were collected over several years and therefore aggregating them together may mask trends in improved survival or incidence over time. The data are also right-censored because many patients are still alive at the end of the data collection period. Additionally estimated survival times are slightly artificially inflated at age 65 due to a discrete stratification of conditional life expectancies (Materials and Methods).

### Esophageal cancer

Esophageal cancer is the sixth most common cause of cancer-related death worldwide, with a 5-year survival rate of less than 20% [58]. Although screening is practiced in a few geographic areas with high-risk populations such as parts of China, endoscopic screening remains expensive and not readily available in many high-risk regions [59]. We perform our analysis for only a subset of population groups for this cancer type as sample sizes in the SEER database are too low for reliable estimates in several cases such as young Asian and Pacific Islanders and Hispanics. We find that, assuming a screen of 75% effectiveness, patients in their 30’s and 40’s are predicted to live up to an additional 20–25 years on average (Fig W in S1 Appendix). For these results age is the most decisive indicator of potential benefit, dwarfing differences by sex and ancestry.

### Kidney cancer

Renal cancers constitute about 2% of all cancer diagnoses and deaths worldwide with incidence rates generally higher in developed countries and rising; there are about 14,000 deaths per year in the US from kidney cancers [60]. Urinary dipsticks are inadequate for screening due to low sensitivity and specificity and CT abdominal imaging or ultrasound are not recommended for population screening due to cost, false positives, incidental findings and low incidence [61]. We find that early screening in 25-34-year-old patients can result in very large survival benefits on average–up to 30 years (Fig 4A). Survival amongst 60–64-year-olds is typically only around 10 years. A notable sex difference is that 60-64-year-old African American females are predicted to survive on average 6 years with screening, whereas their male counterparts are only expected to live for 3 years.

### Liver cancer

Liver cancer is the fourth leading cause of cancer-related death globally and its incidence is growing with estimates of over a million cases per year by 2025 [62]; however, a mass screening trial for liver cancer in China using serum alpha-fetoprotein and ultrasonography did not yield a reduction in liver cancer-specific mortality [63]. Incidence for this cancer type in the SEER database is higher amongst males (Fig W in S1 Appendix). We find that 60-64-year-old men are estimated to benefit on average less than females (about 9 years vs 12 years) across all ancestries with this sex difference especially pronounced in Hispanics and African Americans. Younger patients are predicted to enjoy substantially longer survival: for instance, 35-44-year-old males can expect to gain approximately 25 additional life years as a result of screening.

### Mesothelioma

Mesothelioma is almost always caused by asbestos exposure and currently accounts for about 3,000 deaths per year in the United States, with cases expected to rise in the developing world [64]. Because high-risk cohorts are well known, it is hypothesized that targeted screening could significantly improve survival; however, chest x-rays are insensitive and a combination of CT scans and biomarkers are the subject of continued investigation [65]. Mesothelioma is a rare cancer in SEER owing to its low incidence and as such too little data is available for most population subgroups to perform our analyses apart from Caucasian patients (Fig W in S1 Appendix). Incidence in men is significantly higher than in women, but per patient, women on average can expect to live longer as a result of screening under our modeling assumptions. For instance, 60-64-year-old males are predicted to survive for 7.5 years on average post screening, whereas for females this figure is 11 years. The differential incidence suggests targeted screening may be more appropriate (in a similar manner to that displayed in Fig 2B). This targeting may be done based on professions with high asbestos exposure where such data is available. SEER does not contain such information, but the queuing methodology could be applied on datasets containing such variables.

### Ovarian cancer

There are about 200,000 cases of ovarian cancer globally each year, but this incidence is on the decline; meanwhile 5-year overall survival in the US stands at 45.6% [66]. Transvaginal ultrasound and bimanual pelvic examination have been used in various screening studies, but no mortality benefit has been established for this type of screening [67]. This type of cancer only affects women, so there is no sex stratification (Fig 4B). Our results suggest that identifying this cancer type through a novel and more effective screen in 25-29-year-olds would on average result in overall survival gains of over 30 years under the assumption of 75% effectiveness. In comparison each 65-69-year-old patient would stand to gain about 10 extra years. By age 80 and above the benefit is estimated to be 2 years on average.

### Comparing across cancer types

A comparison of screening programs for the different cancer types is displayed in Fig 4. We show the predicted change in overall survival by cancer type under two example scenarios of screen effectiveness (25% and 75%) for African American and Asian and Pacific Islander men aged 50–54 (Fig 4C and 4D, respectively). Our results suggests that screening for pancreatic cancer would provide the largest survival benefit and kidney cancer the least, which reflects the fact that current survival from the latter is longer than the former, so the potential gains are smaller. The analogous slope chart for other population subgroups is shown in Fig X in S1 Appendix. In all cases pancreatic cancer is a promising target for screening since current survival is poor. Mesothelioma, liver, and esophageal cancer also have low overall survival and therefore may see big gains from successful screening. Ovarian cancer and kidney cancer already have somewhat longer survival (typically between 5–10 years on average), whereas for the other cancer types overall survival without screening for this age group is on average less than 2.5 years. We investigate the survival distributions of cancer patients by cancer type under three scenarios: no screening, screening with 50% effectiveness and with 100% effectiveness (Fig 4E). The fact that ovarian cancer is sometimes diagnosed in very young people (age 20–30) means that under 100% effectiveness some patients could be expected to gain between 40 and 60 life years. Due to mesothelioma incidence being disproportionately concentrated in middle aged and older people, most people screened for this cancer type are estimated to live an additional 10–20 years.

## Discussion

Here we describe how a queuing-theoretic framework can be used as a versatile computational method to generate simple stochastic models to quantify the benefits of screening for cancer and to design optimal allocation and screening strategies. We illustrate the versatility of this modeling approach by discussing example queuing network models that cover a range of medical applications. We demonstrate how the queuing approach permits generalizations that go significantly beyond deterministic compartmental models and Markov chain models, while also providing more detailed answers. The exact results we obtain circumvent the need for simulations entirely and offer a transparent relationship between inputs and outputs of the model. Basic performance analysis of the queuing network models also yields natural and explicit analytical quantifications of the benefits of screening. This finding suggests simple rules for developing optimal screening strategies when resources are scarce and for extending our methodology to factor in cost (as in Fig 2B), which is particularly important in the setting of differential incidence and targeted screening. We apply this modeling framework to datasets from the SEER cancer incidence database with a particular focus on pancreatic cancer.

Although our data applications are based on non-randomized registry data, our approach has utility both in the setting of randomized clinical trial data and non-randomized data. Randomized data is more appropriate when comparing or assessing the effectiveness of different treatments, because non-randomized data has an inherent selection effect since treatments are given based on the patient’s characteristics and not randomly assigned, so any ‘measured benefits’ can be at least partially ascribed to genuine differences between patients. Even in the randomized setting our modeling approach is useful for extrapolating to demand on the population level, including a breakdown by subgroup. Additionally, randomized data combined with our approach can provide a detailed mechanistic understanding linking inputs with outputs, whereas traditional statistical analyses (such as Kaplan-Meier plots) cannot provide such insights. In contrast, registry data, such as that in SEER, can be helpful precisely when no trial data is available to investigate the utility of a new screening technology. Our approach is useful for ascertaining how effective a screening strategy would have to be to make a substantial difference to outcomes like overall survival, well before a trial can be conceived. As an example, consider using circulating tumor DNA readouts from blood tests as an early diagnostic tool in cancer. Registry data can help model how effective such an intervention might be, and therefore indicate whether a trial might be worth pursuing for which cancer type.

Our approach has several modeling assumptions (Materials and Methods), which if changed may change the results or permute the ordering of population subgroups we obtain. Furthermore, uptake in screening programs, the chance of detection and the resultant survival benefit may depend on age, sex, and ancestry, which we do not explicitly consider here, but our methodology can easily be extended to incorporate such scenarios. The focus of most of our results is on the benefit per patient, rather than per screen. The latter depends intimately on differential incidence of cancers including at the population subgroup level, leading to the fact that the per screen benefit ranking may be different from the per patient ranking. This effect is investigated in Fig 2B. Because incidence and treatment standards for cancers vary substantially across countries, an application of results based on US data needs to be refined before being applied to other countries.

Our focus here is on mutually independent waiting times which are naturally modeled by infinite server queues. Alternatively, we can consider finite server queues and the dependent waiting times that they model–see section 2.5 in S1 Appendix. A broad class of such queuing models fit into the framework we describe here and into networks with a mixture of finite and infinite server nodes. Going beyond the BCMP framework means that we lose our analytical results. In this case simulation is the only general-purpose method. For instance, networks with non-Poissonian exogenous arrivals cannot be handled by these methods (just as they cannot by Markov chains). A full discussion of which models are in the scope of the theorem can be found in [37].

Our aim is to illustrate an alternative and potentially useful toolbox through simple examples. With more extensive data one could make much more complicated models that consider all sorts of disease-specific features such as different dynamics for various subtypes and treatments, clinical stages etc. For example, it is easy to incorporate the potential costs of screening such as overdiagnosis and overtreatment into this model, which might simply involve reweighting estimated sojourn times by QALY scores, but it is hard to estimate these effects without a randomized controlled trial. These QALY adjustments may be particularly important when comparing screening strategies and outcomes across cancer types. We omit information of cancer stage in our analysis because SEER does not have consistently defined staging throughout the dataset used.

In sum, we propose that a queuing network-based methodology for evaluating screening approaches can be widely applied in future studies to identify best strategies for clinical implementation.

## Supporting information

S1 AppendixSupporting information containing supplementary methods, tables, and figures.**Fig A. Schematic model of screenable cancer whose early detection confers an overall survival benefit through early treatment.** New instances of undiagnosed cancer appear at rate *η*_1_. The number of such undiagnosed individuals is denoted *N*_1_. These individuals either die, at rate *μ*_1_*r*_10_, are successfully screened and begin early treatment, at rate *μ*_1_*r*_12_, or progress to late-stage symptomatic disease, at rate *μ*_1_*r*_13_. The number of individuals receiving early treatment is denoted *N*_2_ and those receiving late-stage treatment by *N*_3_. The rate of death from the former population is *μ*_2_ and from the latter is *μ*_3_. **Table A. Testing distributional predictions of the model against empirical distributions.** Kolmogorov-Smirnov tests comparing empirical and theoretical distributions predicted by the model. We compare the predicted distributions of $N_{1}^{*}$ and $N_{2}^{*}$ to the empirical distributions from the SEER data and test whether exogenous arrivals are Markovian. Bonferroni adjusted p-values are displayed. **Fig B. Treatment-associated morbidity.** We adjust the survival times by a multiplicative QALY factor representing the decrease in quality of life due to treatment. The more severe the side effects of the treatment, the lower the QALY factor. **Fig C. Treatment-associated mortality.** With probability *p* (given in percentage terms on the x-axis) patients die after one month due to treatment-induced mortality. With probability 1−*p* they get the original, unadjusted survival time distribution. As the risk of mortality increases, the average benefit of early treatment decreases (holding treatment effectiveness constant). **Fig D. Specificity and false positives factored in.** Example circulating tumor DNA pancreatic cancer screening program for Caucasian males aged 55–59. The screen has imperfect specificity so false positives are possible, but confirmatory scans after positive screen results reduce the false positive rate. The x-axis shows different values for the false positive probability and the y-axis the treatment effectiveness. The color shows the net survival benefit from the program, i.e., the survival gains from catching pancreatic cancer early minus the overtreatment of false positives. **Fig E. Difference in overall survival between an effective screening program and no screening for esophageal cancer.** The left end of the dumbbell shows expected survival of unscreened cancer sufferers, the right end shows estimated average survival under a screening program with 100% efficacy. **Fig F. Difference in overall survival between an effective screening program and no screening for kidney cancer.** The left end of the dumbbell shows expected survival of unscreened cancer sufferers, the right end shows estimated average survival under a screening program with 100% efficacy. **Fig G. Difference in overall survival between an effective screening program and no screening for liver cancer.** The left end of the dumbbell shows expected survival of unscreened cancer sufferers, the right end shows estimated average survival under a screening program with 100% efficacy. **Fig H. Difference in overall survival between an effective screening program and no screening for mesothelioma.** The left end of the dumbbell shows expected survival of unscreened cancer sufferers, the right end shows estimated average survival under a screening program with 100% efficacy. There is not enough data to make reliable estimates from many population subgroups. **Fig I. Difference in overall survival between an effective screening program and no screening for ovarian cancer.** The left end of the dumbbell shows expected survival of unscreened cancer sufferers, the right end shows estimated average survival under a screening program with 100% efficacy. **Fig J. Difference in overall survival between an effective screening program and no screening for pancreatic cancer.** The left end of the dumbbell shows expected survival of unscreened cancer sufferers, the right end shows estimated average survival under a screening program with 100% efficacy. **Fig K. Testing the distributional predictions of the BCMP theorem for number of newly diagnosed Asian or Pacific Islander pancreatic cancer sufferers.** We compare the predicted distribution of $N_{1}^{*}$, the number of pancreatic cancer sufferers at a given time diagnosed and awaiting surgery, and the empirical distribution of this quantity estimated from the incidence data. The theorem predicts that $N_{1}^{*}$ should have a Poisson distribution whose mean is found by solving the traffic equations. This process only involves averages of the data, and not details of the data itself. The empirical distribution is also a discrete distribution, but we smooth out the probability mass function of each as it is easier to visualize this way. **Fig L. Testing the distributional predictions of the BCMP theorem for number of newly diagnosed African American pancreatic cancer sufferers.** We compare the predicted distribution of $N_{1}^{*}$, the number of pancreatic cancer sufferers at a given time diagnosed and awaiting surgery, and the empirical distribution of this quantity estimated from the incidence data. The theorem predicts that $N_{1}^{*}$ should have a Poisson distribution whose mean is found by solving the traffic equations. This process only involves averages of the data, and not details of the data itself. The empirical distribution is also a discrete distribution, but we smooth out the probability mass function of each as it is easier to visualize this way. **Fig M. Testing the distributional predictions of the BCMP theorem for number of newly diagnosed Hispanic pancreatic cancer sufferers.** We compare the predicted distribution of $N_{1}^{*}$, the number of pancreatic cancer sufferers at a given time diagnosed and awaiting surgery, and the empirical distribution of this quantity estimated from the incidence data. The theorem predicts that $N_{1}^{*}$ should have a Poisson distribution whose mean is found by solving the traffic equations. This process only involves averages of the data, and not details of the data itself. The empirical distribution is also a discrete distribution, but we smooth out the probability mass function of each as it is easier to visualize this way. **Fig N. Testing the distributional predictions of the BCMP theorem for number of newly diagnosed Caucasian pancreatic cancer sufferers.** We compare the predicted distribution of $N_{1}^{*}$, the number of pancreatic cancer sufferers at a given time diagnosed and awaiting surgery, and the empirical distribution of this quantity estimated from the incidence data. The theorem predicts that $N_{1}^{*}$ should have a Poisson distribution whose mean is found by solving the traffic equations. This process only involves averages of the data, and not details of the data itself. The empirical distribution is also a discrete distribution, but we smooth out the probability mass function of each as it is easier to visualize this way. **Fig O. Testing the distributional predictions of the BCMP theorem for Asian and Pacific Islander pancreatic cancer sufferers receiving surgery.** We compare the predicted distribution of $N_{2}^{*}$, the number of pancreatic cancer sufferers receiving surgery in a given year, and the empirical distribution of this quantity estimated from the incidence data. The theorem predicts that $N_{2}^{*}$ should have a Poisson distribution whose mean is found by solving the traffic equations. This process only involves averages of the data, and not details of the data itself. The empirical distribution is also a discrete distribution, but we smooth out the probability mass function of each as it is easier to visualize this way. **Fig P. Testing the distributional predictions of the BCMP theorem for African American pancreatic cancer sufferers receiving surgery.** We compare the predicted distribution of $N_{2}^{*}$, the number of pancreatic cancer sufferers receiving surgery in a given year, and the empirical distribution of this quantity estimated from the incidence data. The theorem predicts that $N_{2}^{*}$ should have a Poisson distribution whose mean is found by solving the traffic equations. This process only involves averages of the data, and not details of the data itself. The empirical distribution is also a discrete distribution, but we smooth out the probability mass function of each as it is easier to visualize this way. **Fig Q. Testing the distributional predictions of the BCMP theorem for Hispanic pancreatic cancer sufferers receiving surgery.** We compare the predicted distribution of $N_{2}^{*}$, the number of pancreatic cancer sufferers receiving surgery in a given year, and the empirical distribution of this quantity estimated from the incidence data. The theorem predicts that $N_{2}^{*}$ should have a Poisson distribution whose mean is found by solving the traffic equations. This process only involves averages of the data, and not details of the data itself. The empirical distribution is also a discrete distribution, but we smooth out the probability mass function of each as it is easier to visualize this way. **Fig R. Testing the distributional predictions of the BCMP theorem for Caucasian pancreatic cancer sufferers receiving surgery.** We compare the predicted distribution of $N_{2}^{*}$, the number of pancreatic cancer sufferers receiving surgery in a given year, and the empirical distribution of this quantity estimated from the incidence data. The theorem predicts that $N_{2}^{*}$ should have a Poisson distribution whose mean is found by solving the traffic equations. This process only involves averages of the data, and not details of the data itself. The empirical distribution is also a discrete distribution, but we smooth out the probability mass function of each as it is easier to visualize this way. **Fig S. Do pancreatic cancer diagnoses of Asian and Pacific Islanders follow the Markovian assumption for exogenous arrivals required by the BCMP theorem?** We test the model assumption that exogenous arrivals constitute homogeneous Poisson point processes. We fix a time interval of length one year and then ask if the number of arrival increments during that period follow a Poisson distribution with a mean given by the estimated arrival rate multiplied by the time period, one year. This is necessary (but not sufficient) to be a Poisson point process, so if this is violated then certainly the assumption of Markovian arrivals is too. The Poisson distribution and the empirical distribution of the number of patients diagnosed each year are discrete distributions, but we smooth out their probability mass functions for ease of viewing and comparing. The assumed distribution comes solely from averages of the diagnosis data and does not use anything else about the data itself. The empirical distribution is a result of looking at incidence each year broken down by population subgroup. **Fig T. Do pancreatic cancer diagnoses of African Americans follow the Markovian assumption for exogenous arrivals required by the BCMP theorem?** We test the model assumption that exogenous arrivals constitute homogeneous Poisson point processes. We fix a time interval of length one year and then ask if the number of arrival increments during that period follow a Poisson distribution with a mean given by the estimated arrival rate multiplied by the time period, one year. This is necessary (but not sufficient) to be a Poisson point process, so if this is violated then certainly the assumption of Markovian arrivals is too. The Poisson distribution and the empirical distribution of the number of patients diagnosed each year are discrete distributions, but we smooth out their probability mass functions for ease of viewing and comparing. The assumed distribution comes solely from averages of the diagnosis data and does not use anything else about the data itself. The empirical distribution is a result of looking at incidence each year broken down by population subgroup. **Fig U. Do pancreatic cancer diagnoses of Hispanics follow the Markovian assumption for exogenous arrivals required by the BCMP theorem?** We test the model assumption that exogenous arrivals constitute homogeneous Poisson point processes. We fix a time interval of length one year and then ask if the number of arrival increments during that period follow a Poisson distribution with a mean given by the estimated arrival rate multiplied by the time period, one year. This is necessary (but not sufficient) to be a Poisson point process, so if this is violated then certainly the assumption of Markovian arrivals is too. The Poisson distribution and the empirical distribution of the number of patients diagnosed each year are discrete distributions, but we smooth out their probability mass functions for ease of viewing and comparing. The assumed distribution comes solely from averages of the diagnosis data and does not use anything else about the data itself. The empirical distribution is a result of looking at incidence each year broken down by population subgroup. **Fig V. Do pancreatic cancer diagnoses of Caucasians follow the Markovian assumption for exogenous arrivals required by the BCMP theorem?** We test the model assumption that exogenous arrivals constitute homogeneous Poisson point processes. We fix a time interval of length one year and then ask if the number of arrival increments during that period follow a Poisson distribution with a mean given by the estimated arrival rate multiplied by the time period, one year. This is necessary (but not sufficient) to be a Poisson point process, so if this is violated then certainly the assumption of Markovian arrivals is too. The Poisson distribution and the empirical distribution of the number of patients diagnosed each year are discrete distributions, but we smooth out their probability mass functions for ease of viewing and comparing. The assumed distribution comes solely from averages of the diagnosis data and does not use anything else about the data itself. The empirical distribution is a result of looking at incidence each year broken down by population subgroup. **Fig W. Per patient expected lifetime gain of putative screening programs for a variety of cancer types for which there is currently no widespread screening.** (A) Overall predicted survival benefit from a potential esophageal cancer screen of 75% effectiveness stratified by age, sex and ancestry. There is an artificial bump at age 65 due to the discrete stratification of conditional lifetime distributions. (B) Analogous plot for a potential liver cancer screen under the same assumptions. The sample size used to estimate the parameters is given by the point size and no estimate is made where the sample size is too small to be reliable. (C) Analogous plot for mesothelioma screening under the same assumptions. **Fig X. Comparison of the change in overall survival as a result of screening for different cancer types for various population subgroups aged 50–54.** Survival without screening is shown on the left-hand side and the predicted survival with screening is on the right. We display two scenarios: one in which screening has an effectiveness of 25% and another with effectiveness of 75%.(PDF)
