# Supplementary material for: Designing optimal allocations for cancer screening using queuing network models
Source: PLoS Comput Biol. 2022 May 27;18(5):e1010179. doi: 10.1371/journal.pcbi.1010179 (PMC9182689; doi:10.1371/journal.pcbi.1010179)
Supplement: S1 Appendix — Fig A. Schematic model of screenable cancer whose early detection confers an overall survival benefit through early treatment. New instances of undiagnosed cancer appear at rate η1. The number of such undiagnosed individuals is denoted N1. These individuals either die, at rate μ1r10, are successfully screened and begin early treatment, at rate μ1r12, or progress to late-stage symptomatic disease, at rate μ1r13. The number of individuals receiving early treatment is denoted N2 and those receiving late-stage treatment by N3. The rate of death from the former population is μ2 and from the latter is μ3. Table A. Testing distributional predictions of the model against empirical distributions. Kolmogorov-Smirnov tests comparing empirical and theoretical distributions predicted by the model. We compare the predicted distributions of N1* and N2* to the empirical distributions from the SEER data and test whether exogenous arrivals are Markovian. Bonferroni adjusted p-values are displayed. Fig B. Treatment-associated morbidity. We adjust the survival times by a multiplicative QALY factor representing the decrease in quality of life due to treatment. The more severe the side effects of the treatment, the lower the QALY factor. Fig C. Treatment-associated mortality. With probability p (given in percentage terms on the x-axis) patients die after one month due to treatment-induced mortality. With probability 1−p they get the original, unadjusted survival time distribution. As the risk of mortality increases, the average benefit of early treatment decreases (holding treatment effectiveness constant). Fig D. Specificity and false positives factored in. Example circulating tumor DNA pancreatic cancer screening program for Caucasian males aged 55–59. The screen has imperfect specificity so false positives are possible, but confirmatory scans after positive screen results reduce the false positive rate. The x-axis shows different values for the false positive probability and the y-axi [file pcbi.1010179.s001.pdf]

S1 Appendix

Supplementary Methods

1 Queuing Theory

1.1 Introduction and Intuition

Queuing theory is the mathematical study of networks of waiting lines. A queuing model describes a system into which customers arrive, queue up in a buffer, then receive service before exiting the system. Formally, a queue is described by an arrival process (a rule governing how work arrives to the system), a distribution of service times (specifying the amount of work each customer comes bearing) and some number of servers working on the customers. This information is succinctly conveyed by Kendall's $X/Y/Z$ notation [3]. The X specifies the stochastic process of arrivals, Y the service time distribution and Z the number of servers. Consider, for instance, the $M/G/k$ queue. The M tells us that the arrival process is Markovian - specifically that customers arrive at the increments of a homogeneous Poisson point process, the G that the service times follow some general (arbitrary) distribution, and that there are k servers. We mostly focus on infinite server queues - representing situations where customers are served in parallel with unsaturating service capacity and thus have waiting times independent of each other - but we also briefly mention how finite server queues fit into this framework.

More sophisticated models involve networks of queues. The typical picture is that customers arrive exogenously to the network, whereupon they visit some (possibly random) sequence of queues and eventually depart the system. To specify the network requires us to describe each queuing facility (node in the network) and how customers are routed between them.

1.2 Relevant Mathematical Background on Queuing Networks

We briefly introduce infinite server queues and recall some facts about them. Calling an infinite server system a 'queue' is something of a misnomer as no customer ever waits in line (by queue length we mean the number of customers being served at a given time).

The simplest infinite server queuing model is the $M/M/\infty$ queue, where customers arrive according to the increments of a Poisson process of fixed rate λ and their service times are i.i.d. $Exponential(\mu)$ distributed random variables. The integer-valued stochastic process $(N_t; t \geq 0)$ tracking the time evolution of the number of customers in the system is a continuous time irreducible birth-death Markov chain, and as such it admits a unique invariant distribution - in this case a Poisson distribution with mean $\rho := \lambda/\mu$ (see section 5.5.2 of [6] for further details).

The $M/G/\infty$ queue is a generalization of this model, where customer service requirements are i.i.d. and generally distributed according to some distribution F supported on $\mathbb{R}_+$ with finite mean $1/\mu$. In particular we do not assume that the service time distribution is Exponential. Thus the queue length process is no longer a Markov chain since knowledge of the past of the process carries information about the elapsed service times and hence the residual service requirements of customers and is thus informative for the future of the queue length process. Nevertheless, the $M/G/\infty$ queue exhibits the Insensitivity Property, meaning that the equilibrium queue length still follows a $Poisson(\rho)$ distribution [2].

Our cancer screening model is a network of infinite server queues. Exogenous arrivals and customer routing are assumed to be Markovian. The latter means that a customer's state transitions are independent of its past behavior (service times, routing decisions etc.). Routing decisions, arrival times and service times at different queues are all mutually independent. Our network is open, meaning that every customer visits just finitely many queues before exiting the network with probability one.

Suppose the network consists of J queues. Then $\boldsymbol{\eta} = (\eta_1, ..., \eta_J)$ details the rates of the mutually independent external arrival processes into each queue. The $J \times J$ substochastic matrix R has entries listing the various routing probabilities between the queues. We write r_{ij} to be the probability that a customer upon completing service at queue i is routed to queue j and $r_{i0} = 1 - \sum_{j=1}^{J} r_{ij}$ the probability that they exit the network entirely. The service time distribution at queue i is denoted F_i and we write $1/\mu_i$ to be its mean. If service were Markovian, then μ_i would be a bona fide rate, but in general it is not. The aggregate arrival rates (exogenous arrivals plus rerouted customers) into each queue are $\boldsymbol{\lambda} = (\lambda_1, ..., \lambda_J)$. These can be computed by solving the traffic equations $\boldsymbol{\lambda} = \boldsymbol{\eta} + \boldsymbol{\lambda} R$, where all vectors are understood to be row vectors. We write $\rho_j := \lambda_j / \mu_j$ for $j \in \{1, ..., J\}$.

The stochastic process $(\boldsymbol{N}(t); t \geq 0)$, where $\boldsymbol{N}(t) = (N_1(t), ..., N_J(t)) \in \mathbb{Z}_{\geq 0}^J$, tracks the time evolution of the number of customers in each queue in the network. The assumptions listed above ensure that the networks we consider are instances of BCMP networks. A discussion of BCMP networks is beyond the scope of this article, but the interested reader is referred to [1]. The key point for our purposes is that BCMP networks admit product form equilibrium distributions. In our case, this means that the full joint probability distribution of the stationary queue lengths is given by

$$\mathbb{P}(\mathbf{N}^* = \mathbf{n}) = \mathbb{P}(N_1^* = n_1, ..., N_J^* = n_J) = \prod_{j=1}^{J} \frac{e^{-\rho_j} \rho_j^{n_j}}{n_j!},$$

where $\boldsymbol{n} = (n_1, ..., n_J) \in \mathbb{Z}_{\geq 0}^J$ and we use superscript stars to denote stationary quantities. This is a product of independent $Poisson(\rho_j)$ random variables. We henceforth refer to this as the BCMP theorem.

The queues in our network are in general $\cdot/G/\infty$ queues - where the dot means that the arrival process is unspecified. This is a generalization of networks of $\cdot/M/\infty$ queues with Poissonian exogenous traffic, which are mathematically equivalent to Markov processes with the obvious analogous rates. By equivalent, we mean that they satisfy the same Chapman-Kolmogorov equations. In this sense, the queuing network model permits a generalization that goes beyond what can be captured by Markov models.

2 Queuing Network Model Analysis: Distributional Results and Exact Performance Measures

We study an example queuing network model related to cancer screening but such techniques are easily extended to other medical applications and our thorough treatment of this example serves as a simple tutorial into how one can implement them. Our example focuses on a type of cancer that can be screened for and for which early detection improves survival. We first construct and compare two mathematical models. We start by analyzing a compartmental model and then show how this can be upgraded to a queuing network model. We quantify the survival benefits conferred by screening by analyzing the stochastic model.

2.1 A Motivating Deterministic Model Analysis

We model a type of cancer that can be screened for and for which early detection improves survival. We track the number of individuals who have developed the cancer but are as yet undiagnosed (N_1), the number where it has been detected early and therefore is being treated early (N_2), and the number who were not successfully screened and have late-stage symptomatic cancer and are thus receiving treatment at an advanced stage (N_3) - see Fig A. Note that we drop the explicit time dependence to lighten notation. We do not track the number of

healthy individuals. Augmenting the model to include this does not make the analysis harder, but somewhat needlessly increases the number of parameters.

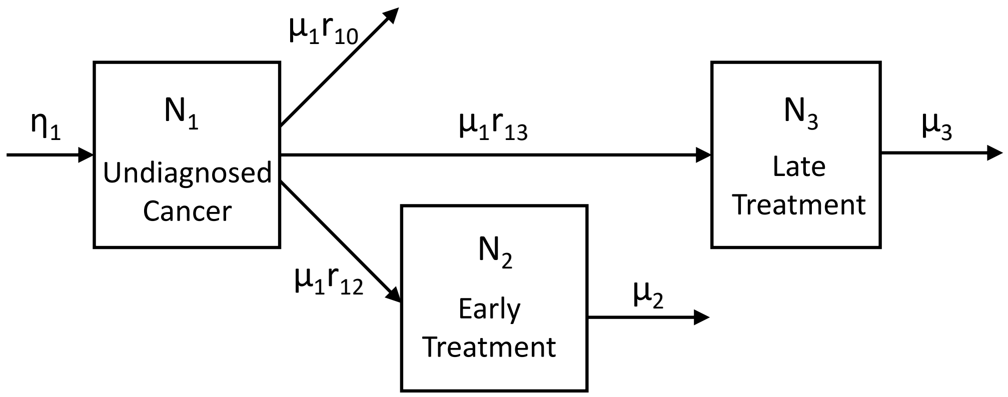

Fig A – Schematic model of screenable cancer whose early detection confers an overall survival benefit through early treatment. New instances of undiagnosed cancer appear at rate η_1. The number of such undiagnosed individuals is denoted N_1. These individuals either die, at rate $\mu_1 r_{10}$, are successfully screened and begin early treatment, at rate $\mu_1 r_{12}$, or progress to late-stage symptomatic disease, at rate $\mu_1 r_{13}$. The number of individuals receiving early treatment is denoted N_2 and those receiving late-stage treatment by N_3. The rate of death from the former population is μ_2 and from the latter is μ_3.

New individuals that develop cancer (but are undiagnosed) arrive at rate η_1. These individuals die at rate μ_{10}, are successfully screened and enter early treatment at rate μ_{12} and progress to late stage symptomatic cancer and start receiving treatment at an advanced stage of disease at rate μ_{13}, hence the total rate of exit of an individual from the first compartment is given by the decomposition $\mu_1 = \mu_{10} + \mu_{12} + \mu_{13}$ and note the fraction taking each path is

$$r_{10} = \frac{\mu_{10}}{\mu_{10} + \mu_{12} + \mu_{13}}, \quad r_{12} = \frac{\mu_{12}}{\mu_{10} + \mu_{12} + \mu_{13}}, \quad \text{and} \quad r_{13} = \frac{\mu_{13}}{\mu_{10} + \mu_{12} + \mu_{13}}.$$

Rearranging, we find that the rate of death from the first compartment is $\mu_1 r_{10}$, the rate of being detected by a successful screen and entering early treatment is $\mu_1 r_{12}$, and the rate of progressing to late-stage symptomatic disease and entering late treatment is $\mu_1 r_{13}$. Individuals receiving early treatment eventually die (whether from competing risks or the cancer itself) at rate μ_2, while individuals receiving late-stage treatment die at rate μ_3. Thus the system evolves according to the following linear set of coupled ODEs:

$$\frac{dN_1}{dt} = \eta_1 - \mu_1 N_1, \qquad \frac{dN_2}{dt} = \mu_1 r_{12} N_1 - \mu_2 N_2, \qquad \frac{dN_3}{dt} = \mu_1 r_{13} N_1 - \mu_3 N_3.$$

It is straightforward to find the steady state solution of this system. In practice, we almost always observe this system in its steady state. Viewed over long timescales it may not be stationary (imagine changes in life expectancy or lifestyle over generations) or indeed over very short timescales (imagine the rate of patients arriving for a screen at different times of day), but over relevant timescales it is safe to assume it is locally stationary. An exception to this would include a rare event like a nuclear disaster which drastically changes the incidence of cancer over a short timescale. Denoting steady state quantities with superscript stars, we find:

$$N_1^* = \frac{\eta_1}{\mu_1}, \qquad N_2^* = \frac{\eta_1 r_{12}}{\mu_2}, \qquad N_3^* = \frac{\eta_1 r_{13}}{\mu_3}. \tag{1}$$

2.2 Translation into a Stochastic Queuing Network Model

We now translate this model into a queuing network model which we proceed to analyze. To do so, we draw an analogy between the number of individuals in certain disease states (those represented by compartments

above) and the number of customers in each of a collection of queues arranged into a network. Each queue represents a particular disease state and so its occupancy tracks the number of individuals in that state over time. An arrival of a new customer into a given queue, corresponds to an increase in the number of individuals in that state. Conversely, a service completion and departure of a customer from a given queue, represents an individual leaving that state. A customer being routed from one queue to another represents an individual making a state transition. So the service requirement of a customer can be seen as the waiting time of an individual in that state.

In this example no individual waits for another to change their own state. For instance, the time it takes for an individual receiving treatment to die does not depend on other individuals. This corresponds to having an infinite number of servers in the late treatment queue - regardless of the occupancy of the system, a customer's sojourn time in the queue is precisely their own service time. With an infinite number of servers, arriving customers never need to wait, there is always an available server to process them. This logic underlies why all of the queues in this example have infinitely many servers.

Replacing compartments with queues and deterministic rates with random processes yields the analogous queuing network model and the notation carries over in the obvious way, but now $N_i(t)$ is viewed as a stochastic queue length process, η_i is the rate of a homogeneous Poisson point process into queue i, the r_{ij} become Markovian routing probabilities, and μ_i is no longer a rate, but instead is defined to be the reciprocal of the mean service time at queue i.

2.3 Aggregate Arrival Rates and Stationary Distributions

We shall solve the traffic equations to find the aggregate arrival rates and then write down the full joint equilibrium probability distribution of the network. Then basic performance analysis allows us to quantify the benefit of screening by comparing it to a network where this intervention is absent. The traffic equations are parameterized by

$$R = \begin{bmatrix} 0 & r_{12} & r_{13} \\ 0 & 0 & 0 \\ 0 & 0 & 0 \end{bmatrix}, \text{ and } \boldsymbol{\eta} = \begin{bmatrix} \eta_1 \\ 0 \\ 0 \end{bmatrix}. \text{ Hence, } \boldsymbol{\lambda} = \begin{bmatrix} \lambda_1 \\ \lambda_2 \\ \lambda_3 \end{bmatrix} = \begin{bmatrix} \eta_1 \\ \eta_1 r_{12} \\ \eta_1 r_{13} \end{bmatrix}.$$

Invoking the BCMP theorem yields the following generalization of equation (1)

$$N_1^* \sim Poisson\left(\frac{\eta_1}{\mu_1}\right), \quad N_2^* \sim Poisson\left(\frac{\eta_1 r_{12}}{\mu_2}\right), \quad N_3^* \sim Poisson\left(\frac{\eta_1 r_{13}}{\mu_3}\right). \tag{2}$$

In expectation this matches what we found for the deterministic model, but now we have a much fuller description. This distributional characterization is useful when forecasting fluctuations in demand such as deciding the number of beds, staffing levels etc. which depend on the distribution of these quantities beyond their averages.

2.4 Performance Analysis and Quantifying the Benefits of Screening

We now quantify the benefits of the intervention. There are many possible metrics that one could use. The simplest thing to do is to note what fraction of patients are successfully screened. This is just r_{12} and so this tells you the gains made by increasing μ_{12}, which corresponds to better detection, diagnosis or reducing the time between diagnosis and treatment.

Although knowing the number of individuals with cancer in early or late-stage treatment may be useful for allocating resources, it is not a helpful way to think about the benefits of interventions. This is because, holding all else equal, improving the screen results in an increase in the typical number of cancer patients. This of course does not mean that more people are getting sick (that depends only on η_1), it simply means that individuals are living longer and so there are typically more alive with cancer at any given time.

A more suitable metric is the average residual lifetime. This corresponds to the expected sojourn time of a customer in the network. We denote the sojourn time in the network at stationarity by W^*. To calculate the expected sojourn times of customers in various queues, or the network as a whole, one can use Little's Law [4]. This states that the stationary long run average number of customers in the system equals the (long run) average arrival rate, multiplied by the average time spent by a customer in the system. Writing N^* to denote the stationary number of customers in the entire network, linearity of expectation yields

$$\mathbb{E}(N^*) = \mathbb{E}(N_1^*) + \mathbb{E}(N_2^*) + \mathbb{E}(N_3^*) = \eta_1 \left[\frac{1}{\mu_1} + \frac{r_{12}}{\mu_2} + \frac{r_{13}}{\mu_3} \right]. \tag{3}$$

Hence by Little's Law,

$$\mathbb{E}(W^*) = \frac{\mathbb{E}(N^*)}{\eta_1} = \frac{1}{\mu_1} + \frac{r_{12}}{\mu_2} + \frac{r_{13}}{\mu_3} = \frac{1}{\mu_{10} + \mu_{12} + \mu_{13}} \left[1 + \frac{\mu_{12}}{\mu_2} + \frac{\mu_{13}}{\mu_3} \right]. \tag{4}$$

We compare this quantity to its analog in a network without screening. In such a network the early treatment queue and all parameters with a subscript $'2'$ are absent. This changes the routing probabilities and μ_1. All the remaining parameters are the same. The analysis for this network proceeds in exactly the same way, so we omit the details. Writing W^*_{with} for the sojourn time in the network with screening and $W^*_{without}$ for the network without and using linearity yields the expected residual lifetime gain (denoted G^*):

$$\mathbb{E}(G^*) = \mathbb{E}(W^*_{with}) - \mathbb{E}(W^*_{without}) = \frac{1}{\mu_{10} + \mu_{12} + \mu_{13}} \left[1 + \frac{\mu_{12}}{\mu_2} + \frac{\mu_{13}}{\mu_3} \right] - \frac{1}{\mu_{10} + \mu_{13}} \left[1 + \frac{\mu_{13}}{\mu_3} \right]. \tag{5}$$

Comparing a new screening technology to an existing one requires computing the difference in the expected sojourn times of two identically structured networks with different parameters. To factor in quality adjustments to survival times one can reweight the waiting times by some QALY multiplier between zero and one for each queue.

Designing a screening strategy depends on whether multiple screens on the same individual are independent given their disease status. If they are conditionally independent, then multiple rounds of testing may be justified to reduce error rates (though the benefits of this should be weighed against screening lower risk groups for the first time if there are scarcity constraints). If they are not, then it becomes important to determine to what extent the outcomes are correlated. For instance, if they are perfectly correlated (the screen gives the same result every time on the same patient given their disease state), then there is nothing to be gained by rescreening. To model repeated screens one could have a series of queues representing each screen in succession in a program, or change the routing matrix so that some proportion of people are rerouted to the undiagnosed node. Different population subgroups could have different network architecture depending on the number of screens and time interval between screens.

2.5 Complex Networks

We now demonstrate how the same approach is easily extended to more complicated networks. This toy example is a hybrid network of infinite and finite server queues which still falls within the scope of the BCMP theorem. We briefly mention the additional background queuing theory that we need. The $M/M/1$ queue is a single server queue where customers are served in first-come-first-served order with Markovian arrivals and a Markovian service time distribution. If the arrival rate, λ, is less than the service rate, μ, then the stationary number of customers in the queue follows a (pessimistic) Geometric distribution with parameter $1 - \rho$, where $\rho := \lambda/\mu$ [2].

We model a subgroup of the population with certain characteristics in the same geographical location who have access to the same hospital and oncologist. We imagine that this population subgroup is routinely tested for circulating tumor DNA to catch pre-symptomatic cancers earlier.

We track the number of individuals over time in this population subgroup with pre-symptomatic disease, denoted $N_1(t)$. Once circulating tumor DNA is detected, these individuals wait for an appointment to be

scanned - the number of such individuals is given by the stochastic process $N_2(t)$. After the scan most patients wait for an appointment with the oncologist, the number of which is $N_3(t)$. A fraction of these patients are deemed not to need treatment and are just monitored - the number in this state at time t is $N_4(t)$. Some patients are treated for cancer after seeing the oncologist - the number of which we denote $N_5(t)$.

New cases of pre-symptomatic disease develop in the relevant population subgroup according to a homogeneous Poisson point process of rate η_1. These cases are detected by a circulating tumor DNA test and sent to be scanned with probability r_{12} or die of competing risks first with probability r_{10}. With probability r_{22} a scan is inconclusive and the patient has to be scanned again. With probability r_{23} the patient makes an appointment with the oncologist and with probability r_{20} the patient dies while waiting for the scan appointment (whether by competing risks or otherwise). After seeing the oncologist the patient is either deemed to just require monitoring with probability r_{34} or treatment with probability r_{35}, or dies before seeing the oncologist with probability r_{30}. A patient being monitored is eventually referred back to the oncologist with probability r_{43} or dies with probability r_{40}. A patient who receives treatment will eventually die.

There is just one scanner and one oncologist of the relevant type at the hospital, represented by first-come-first-served single server queues. Assume Markovian service times in these queues with mean service times $1/\mu_2$ and $1/\mu_3$ respectively. All other queues are infinite server facilities - representing the fact that capacity is great enough to monitor or treat all patients in parallel and that patients independently develop pre-symptomatic cancer. These queues have arbitrary service time distributions with mean denoted by $1/\mu_i$ for queue $i = 1, 4, 5$.

In this case

$$R = \begin{bmatrix} 0 & r_{12} & 0 & 0 & 0 \\ 0 & r_{22} & r_{23} & 0 & 0 \\ 0 & 0 & 0 & r_{34} & r_{35} \\ 0 & 0 & r_{43} & 0 & 0 \\ 0 & 0 & 0 & 0 & 0 \end{bmatrix}, \text{ and } \boldsymbol{\eta} = \begin{bmatrix} \eta_1 \\ 0 \\ 0 \\ 0 \\ 0 \end{bmatrix}. \text{ Hence, } \boldsymbol{\lambda} = \begin{bmatrix} \lambda_1 \\ \lambda_2 \\ \lambda_3 \\ \lambda_4 \\ \lambda_5 \end{bmatrix} = \begin{bmatrix} \eta_1 \\ \frac{r_{12}\eta_1}{1-r_{22}} \\ \frac{r_{12}r_{23}\eta_1}{(1-r_{22})(1-r_{43}r_{34})} \\ \frac{r_{12}r_{23}r_{34}\eta_1}{(1-r_{22})(1-r_{43}r_{34})} \\ \frac{r_{12}r_{23}r_{35}\eta_1}{(1-r_{22})(1-r_{43}r_{34})} \end{bmatrix}.$$

Simplifying notation by writing $\rho_i := \lambda_i/\mu_i$ we invoke the BCMP theorem (which also applies to $\cdot/M/1$ first-come-first-served queues [1]) yielding

$$N_1^* \sim Poisson\,(\rho_1)\,, N_2^* \sim Geom\,(1-\rho_2)\,, N_3^* \sim Geom\,(1-\rho_3)\,, N_4^* \sim Poisson\,(\rho_4)\,, N_5^* \sim Poisson\,(\rho_5)\,. \quad (6)$$

Therefore, the expected number of customers in the network is

$$\begin{aligned} \mathbb{E}(N^*) &= \mathbb{E}(N_1^*) + \mathbb{E}(N_2^*) + \mathbb{E}(N_3^*) + \mathbb{E}(N_4^*) + \mathbb{E}(N_5^*) \\ &= \rho_1 + \frac{\rho_2}{1-\rho_2} + \frac{\rho_3}{1-\rho_3} + \rho_4 + \rho_5. \end{aligned} \quad (7)$$

Hence by Little's Law,

$$\mathbb{E}(W^*) = \frac{\mathbb{E}(N^*)}{\eta_1}. \quad (8)$$

One can compare survival with and without the circulating tumor DNA diagnostic test that allows for earlier detection of pre-symptomatic cancer. This involves changing a number of parameters in the network as patients will on average be diagnosed at a later stage. Once one has set up and analyzed a model of this scenario in the same way, then the expected gain from the intervention is computed as

$$\mathbb{E}(G^*) = \mathbb{E}(W^*_{with}) - \mathbb{E}(W^*_{without}).$$

Two further useful performance analysis metrics follow from operational laws similar to Little's Law (see chapters 6.8 and 6.10 of [2]). These are the average number of visits a customer makes to a given queue and the average total service demand made at that node by a (possibly) cycling customer. In terms of the application, we can ask how many times on average an individual is scanned or sees the oncologist and how much total time

they spend on average waiting. These quantities are useful for insurance companies when estimating costs and for hospitals when allocating resources to improve the patient experience.

Let W_i^* denote the sojourn time of a customer at queue i at stationarity, V_i^* the number of visits a customer makes to node i and D_i^* the total demand made by a customer from node i. Define $\theta := \sum_i \eta_i$ to be the total rate at which work arrives externally to the network. Then we have that

$$\mathbb{E}(V_i^*) = \frac{\lambda_i}{\theta} \quad \text{and} \quad \mathbb{E}(D_i^*) = \mathbb{E}(V_i^*)\mathbb{E}(W_i^*).$$

In this case the expected number of times an individual is scanned and sees the oncologist are given by

$$\mathbb{E}(V_2^*) = \frac{\lambda_2}{\eta_1} = \frac{r_{12}}{1 - r_{22}} \quad \text{and} \quad \mathbb{E}(V_3^*) = \frac{\lambda_3}{\eta_1} = \frac{r_{12}r_{23}}{(1 - r_{22})(1 - r_{43}r_{34})} \quad \text{respectively,} \tag{9}$$

and the expected total time spent waiting for each is

$$\mathbb{E}(D_2^*) = \frac{\lambda_2}{\eta_1} \cdot \frac{\mathbb{E}(N_2^*)}{\lambda_2} = \frac{\rho_2}{\eta_1(1 - \rho_2)} \quad \text{and} \quad \mathbb{E}(D_3^*) = \frac{\lambda_3}{\eta_1} \cdot \frac{\mathbb{E}(N_3^*)}{\lambda_3} = \frac{\rho_3}{\eta_1(1 - \rho_3)} \quad \text{respectively.} \tag{10}$$

2.6 Extensions

The BCMP theorem extends to many other queuing models, including Markovian multiserver queues and other single server queues. Choosing a model that falls outside the scope of the BCMP theorem means that we lose the distributional results. One such example is the case of non-Poissonian exogenous arrivals. This rules out instances when disease incidence changes very rapidly over a short timescale. A quick test to establish whether traffic is not Poissonian is to look at the number of arrivals in periods of some fixed length and then calculate the sample variance of these numbers, divided by the sample mean. If the result is significantly away from unity, then the Poisson assumption is a poor one. In such situations we call the arrival process over or under dispersed. There is a large body of work on networks of queues with more general arrival processes (see for instance [5]), but this is beyond the scope of this article.

An alternative approach to simulation when the model is not amenable to exact analysis is to use a limit theorem. When rescaling time and space, taking a formal mathematical scaling limit, one observes, under very general settings, a stochastic differential equation that describes the abundances of each disease state. Specifically, for the networks of infinite server queues we considered, one obtains a multidimensional reflected Ornstein-Uhlenbeck process in the non-negative orthant [7]. An advantage of using a limit theorem rather than starting with an SDE model, is that one can appreciate where the randomness comes from at the microscopic scale, rather than simply adding a catch-all artificial white noise term.

References

[1] Baskett,F., Chandy,K., Muntz,R., Palacios,F. (1975). Open, closed and mixed networks of queues with different classes of customers. *Journal of the ACM*, 22(2):248-260.

[2] Harchol-Balter,M. (2013). *Performance Modeling and Design of Computer Systems: Queueing Theory in Action*, Cambridge University Press.

[3] Kendall,D. (1953). Stochastic Processes Occurring in the Theory of Queues and their Analysis by the Method of the Imbedded Markov Chain. *Annals of Mathematical Statistics*, 24(3):338-354.

[4] Little,J. (1961). A Proof for the Queuing Formula $L = \lambda W$. *Operations Research*, 9(3):383-387.

[5] Massey,W., Whitt,W. (1993). Networks of infinite-server queues with nonstationary Poisson input. *Queueing Systems*, 13:183-250.

[6] Mitrani,I. (1998). *Probabilistic Modelling*, Cambridge University Press.

[7] Whitt,W. (1982). On the heavy-traffic limit theorem for $GI/G/\infty$ queues. *Advances in Applied Probability*, 14:171-190.

Supplementary Tables

**Ancestry**	**Sex**	**Age**	N_1^* **adjusted p-value**	N_2^* **adjusted p-value**	**Arrival Process adjusted p-value**
African American	Female	40-44	>0.99	>0.99	>0.99
African American	Female	45-49	>0.99	>0.99	>0.99
African American	Female	50-54	>0.99	>0.99	>0.99
African American	Female	55-59	>0.99	>0.99	>0.99
African American	Female	60-64	>0.99	>0.99	0.98
African American	Female	65-69	>0.99	>0.99	>0.99
African American	Female	70-74	>0.99	>0.99	>0.99
African American	Female	75-79	>0.99	>0.99	>0.99
African American	Female	80-84	>0.99	>0.99	>0.99
African American	Male	40-44	>0.99	>0.99	>0.99
African American	Male	45-49	>0.99	>0.99	>0.99
African American	Male	50-54	>0.99	>0.99	>0.99
African American	Male	55-59	>0.99	>0.99	>0.99
African American	Male	60-64	>0.99	>0.99	>0.99
African American	Male	65-69	>0.99	>0.99	>0.99
African American	Male	70-74	>0.99	>0.99	>0.99
African American	Male	75-79	>0.99	>0.99	>0.99
African American	Male	80-84	>0.99	>0.99	>0.99
Asian/Pacific	Female	45-49	>0.99	>0.99	>0.99
Asian/Pacific	Female	50-54	>0.99	>0.99	>0.99
Asian/Pacific	Female	55-59	>0.99	0.05	>0.99
Asian/Pacific	Female	60-64	>0.99	>0.99	>0.99
Asian/Pacific	Female	65-69	>0.99	>0.99	>0.99
Asian/Pacific	Female	70-74	>0.99	>0.99	>0.99
Asian/Pacific	Female	75-79	>0.99	>0.99	>0.99
Asian/Pacific	Female	80-84	>0.99	>0.99	>0.99
Asian/Pacific	Male	40-44	>0.99	>0.99	>0.99
Asian/Pacific	Male	45-49	>0.99	>0.99	>0.99
Asian/Pacific	Male	50-54	>0.99	>0.99	>0.99
Asian/Pacific	Male	55-59	>0.99	>0.99	>0.99
Asian/Pacific	Male	60-64	>0.99	>0.99	>0.99
Asian/Pacific	Male	65-69	>0.99	0.89	>0.99
Asian/Pacific	Male	70-74	>0.99	>0.99	>0.99
Asian/Pacific	Male	75-79	>0.99	>0.99	>0.99
Asian/Pacific	Male	80-84	>0.99	>0.99	>0.99
Caucasian	Female	30-34	>0.99	>0.99	>0.99
Caucasian	Female	35-39	>0.99	>0.99	>0.99
Caucasian	Female	40-44	>0.99	>0.99	>0.99
Caucasian	Female	45-49	>0.99	>0.99	>0.99
Caucasian	Female	50-54	>0.99	>0.99	>0.99
Caucasian	Female	55-59	>0.99	>0.99	>0.99
Caucasian	Female	60-64	>0.99	0.66	0.56
Caucasian	Female	65-69	0.24	0.01	0.06

Caucasian	Female	70-74	>0.99	>0.99	>0.99
Caucasian	Female	75-79	>0.99	>0.99	>0.99
Caucasian	Female	80-84	>0.99	>0.99	>0.99
Caucasian	Male	30-34	>0.99	>0.99	>0.99
Caucasian	Male	35-39	>0.99	>0.99	>0.99
Caucasian	Male	40-44	>0.99	>0.99	>0.99
Caucasian	Male	45-49	>0.99	>0.99	>0.99
Caucasian	Male	50-54	>0.99	>0.99	>0.99
Caucasian	Male	55-59	>0.99	>0.99	>0.99
Caucasian	Male	60-64	0.28	0.48	0.10
Caucasian	Male	65-69	0.15	0.10	0.02
Caucasian	Male	70-74	0.96	0.15	0.10
Caucasian	Male	75-79	>0.99	>0.99	>0.99
Caucasian	Male	80-84	>0.99	>0.99	>0.99
Hispanic	Female	40-44	>0.99	0.64	>0.99
Hispanic	Female	45-49	>0.99	0.06	>0.99
Hispanic	Female	50-54	>0.99	>0.99	>0.99
Hispanic	Female	55-59	>0.99	>0.99	>0.99
Hispanic	Female	60-64	>0.99	>0.99	0.55
Hispanic	Female	65-69	>0.99	0.74	>0.99
Hispanic	Female	70-74	>0.99	>0.99	>0.99
Hispanic	Female	75-79	>0.99	>0.99	>0.99
Hispanic	Female	80-84	>0.99	>0.99	>0.99
Hispanic	Male	35-39	>0.99	>0.99	>0.99
Hispanic	Male	40-44	>0.99	>0.99	>0.99
Hispanic	Male	45-49	>0.99	>0.99	>0.99
Hispanic	Male	50-54	>0.99	>0.99	>0.99
Hispanic	Male	55-59	>0.99	>0.99	>0.99
Hispanic	Male	60-64	>0.99	>0.99	>0.99
Hispanic	Male	65-69	>0.99	0.08	>0.99
Hispanic	Male	70-74	>0.99	>0.99	>0.99
Hispanic	Male	75-79	>0.99	>0.99	>0.99
Hispanic	Male	80-84	>0.99	>0.99	>0.99

Table A – Testing distributional predictions of the model against empirical distributions. Kolmogorov-Smirnov tests comparing empirical and theoretical distributions predicted by the model. We compare the predicted distributions of N_1^* and N_2^* to the empirical distributions from the SEER data and test whether exogenous arrivals are Markovian. Bonferroni adjusted p-values are displayed.

Supplementary Figures

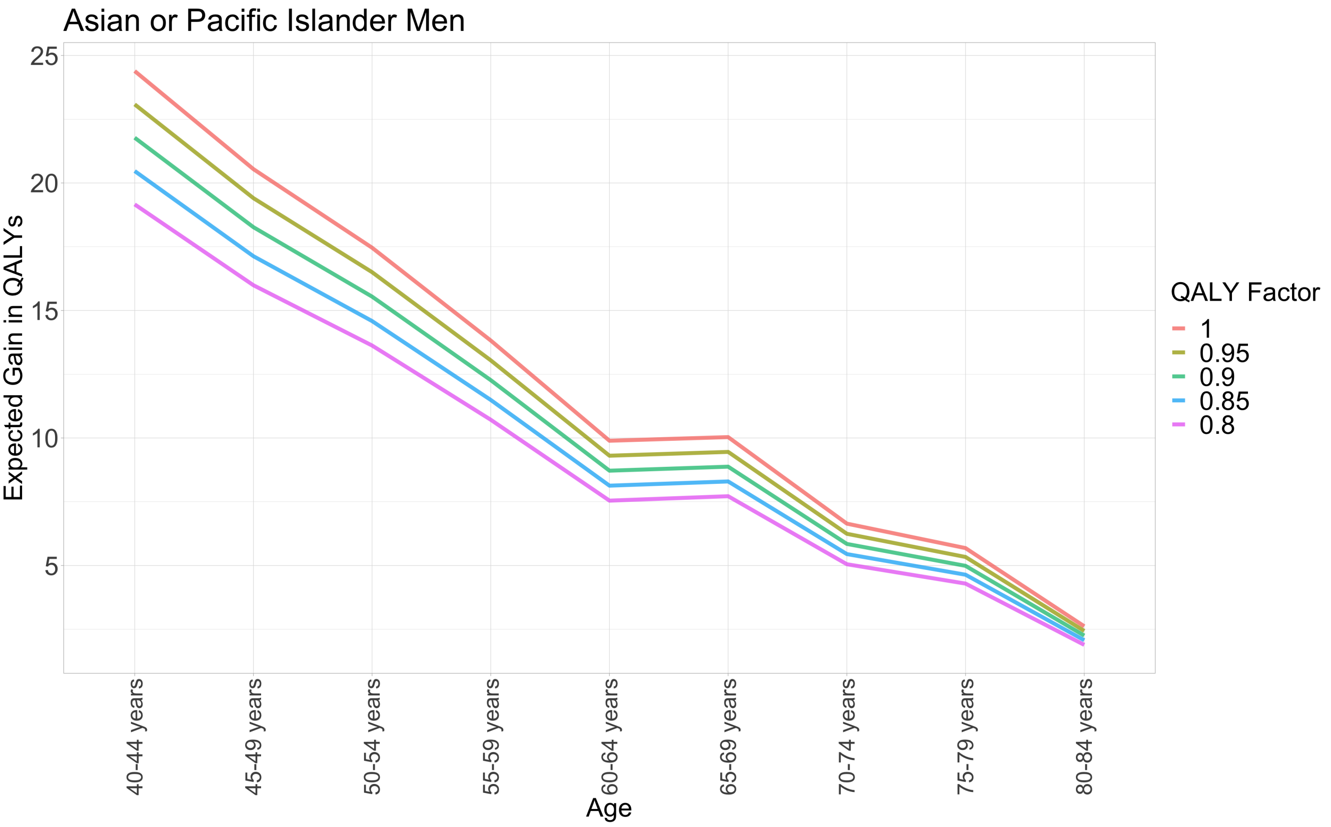

Fig B – Treatment-associated morbidity. We adjust the survival times by a multiplicative QALY factor representing the decrease in quality of life due to treatment. The more severe the side effects of the treatment, the lower the QALY factor.

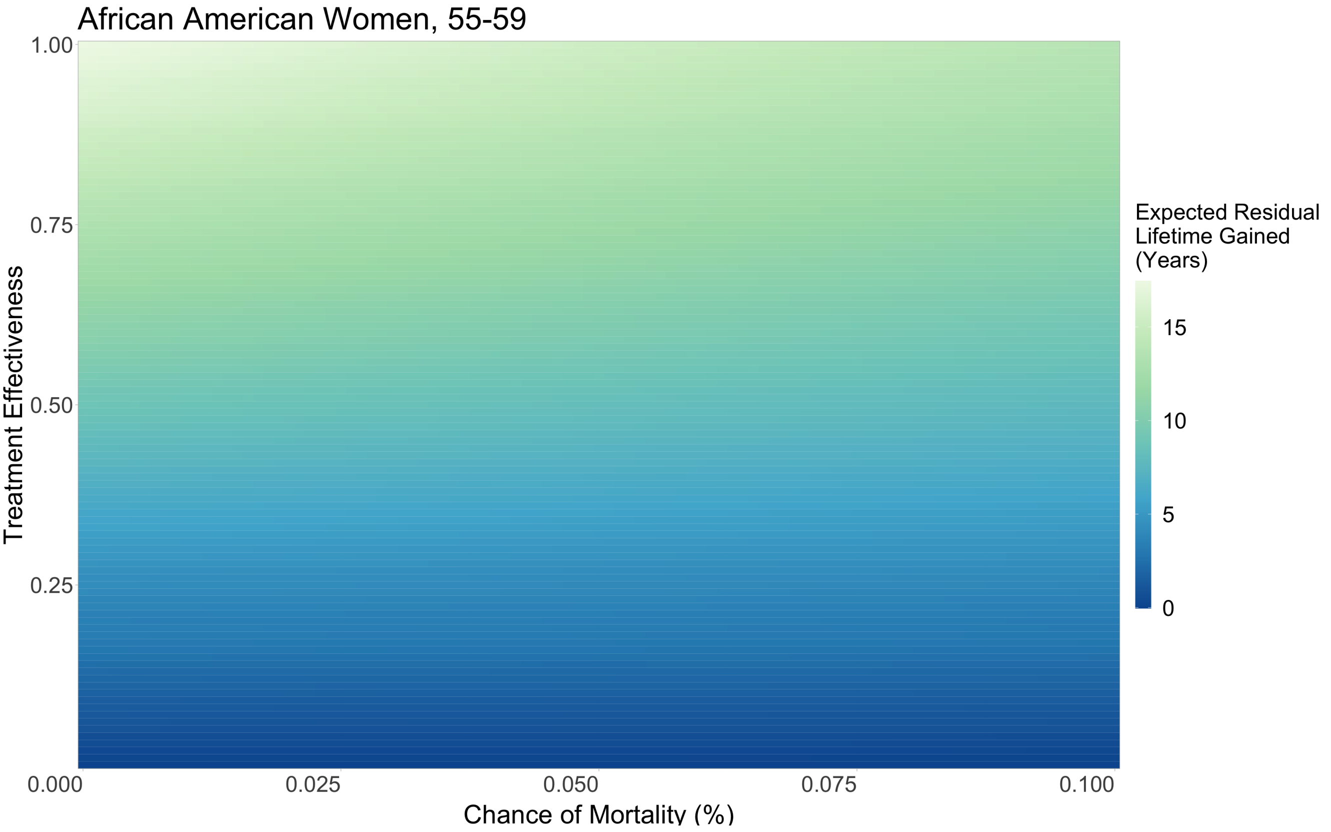

Fig C – Treatment-associated mortality. With probability p (given in percentage terms on the x-axis) patients die after one month due to treatment-induced mortality. With probability $1-p$ they get the original, unadjusted survival time distribution. As the risk of mortality increases, the average benefit of early treatment decreases (holding treatment effectiveness constant).

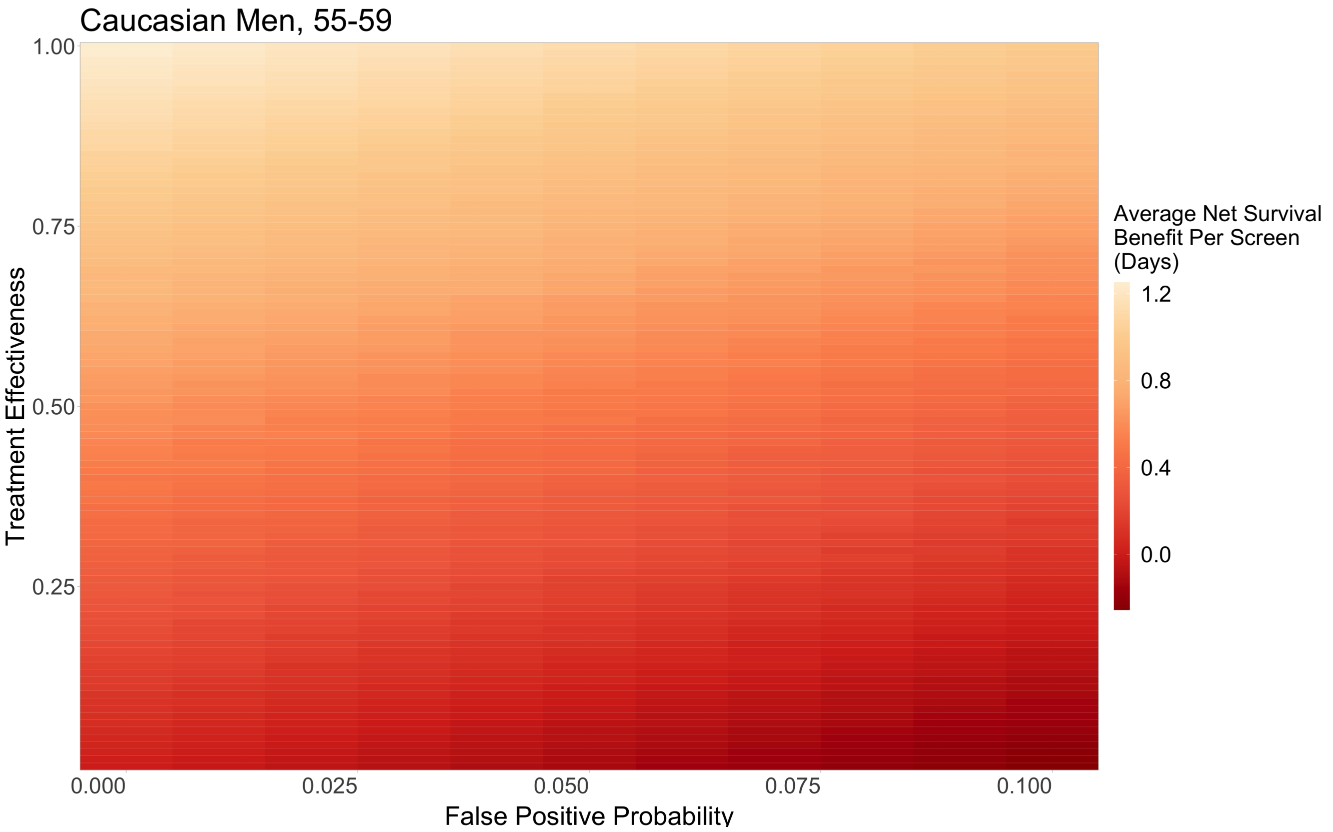

Fig D – Specificity and false positives factored in. Example circulating tumor DNA pancreatic cancer screening program for Caucasian males aged 55-59. The screen has imperfect specificity so false positives are possible, but confirmatory scans after positive screen results reduce the false positive rate. The x-axis shows different values for the false positive probability and the y-axis the treatment effectiveness. The color shows the net survival benefit from the program, i.e., the survival gains from catching pancreatic cancer early minus the overtreatment of false positives.

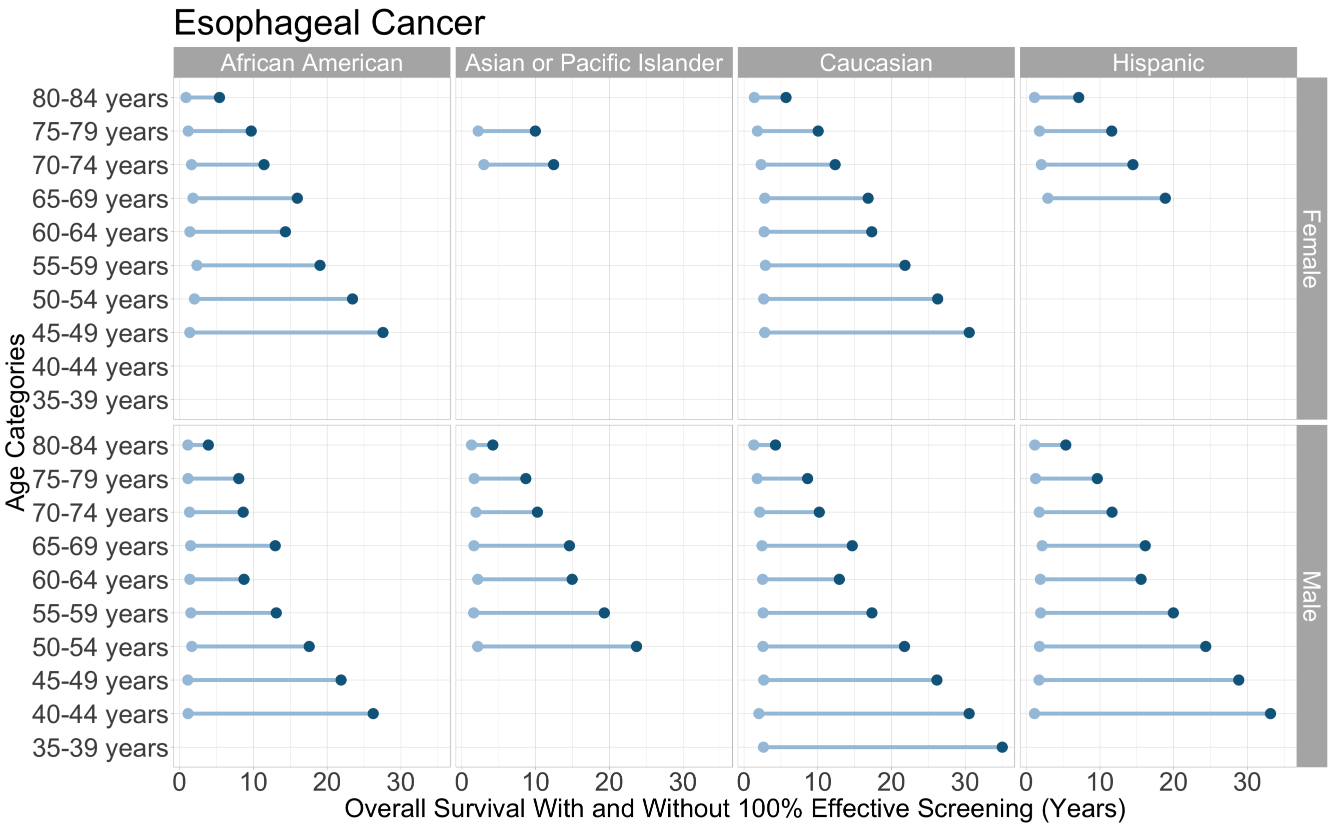

Fig E – Difference in overall survival between an effective screening program and no screening for esophageal cancer. The left end of the dumbbell shows expected survival of unscreened cancer sufferers, the right end shows estimated average survival under a screening program with 100% efficacy.

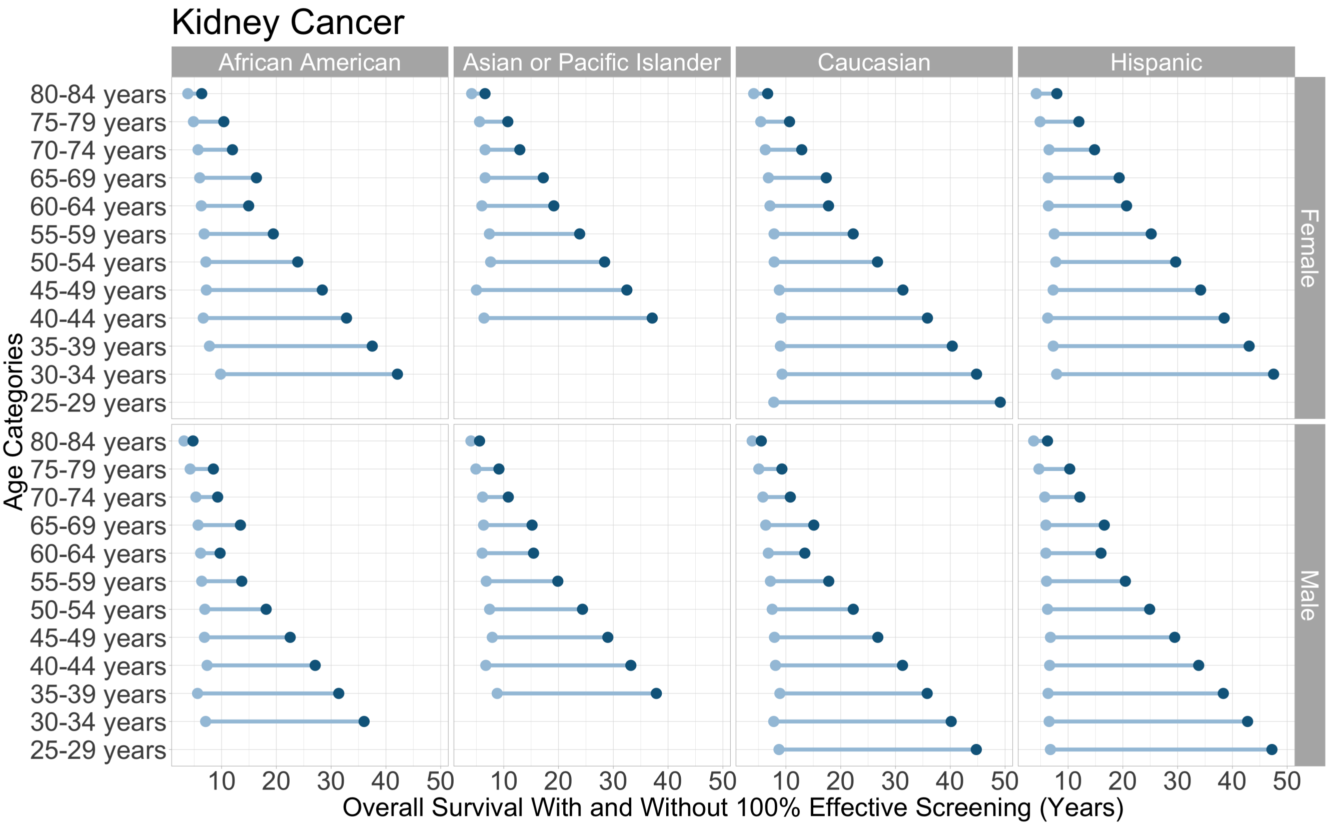

Fig F – Difference in overall survival between an effective screening program and no screening for kidney cancer. The left end of the dumbbell shows expected survival of unscreened cancer sufferers, the right end shows estimated average survival under a screening program with 100% efficacy.

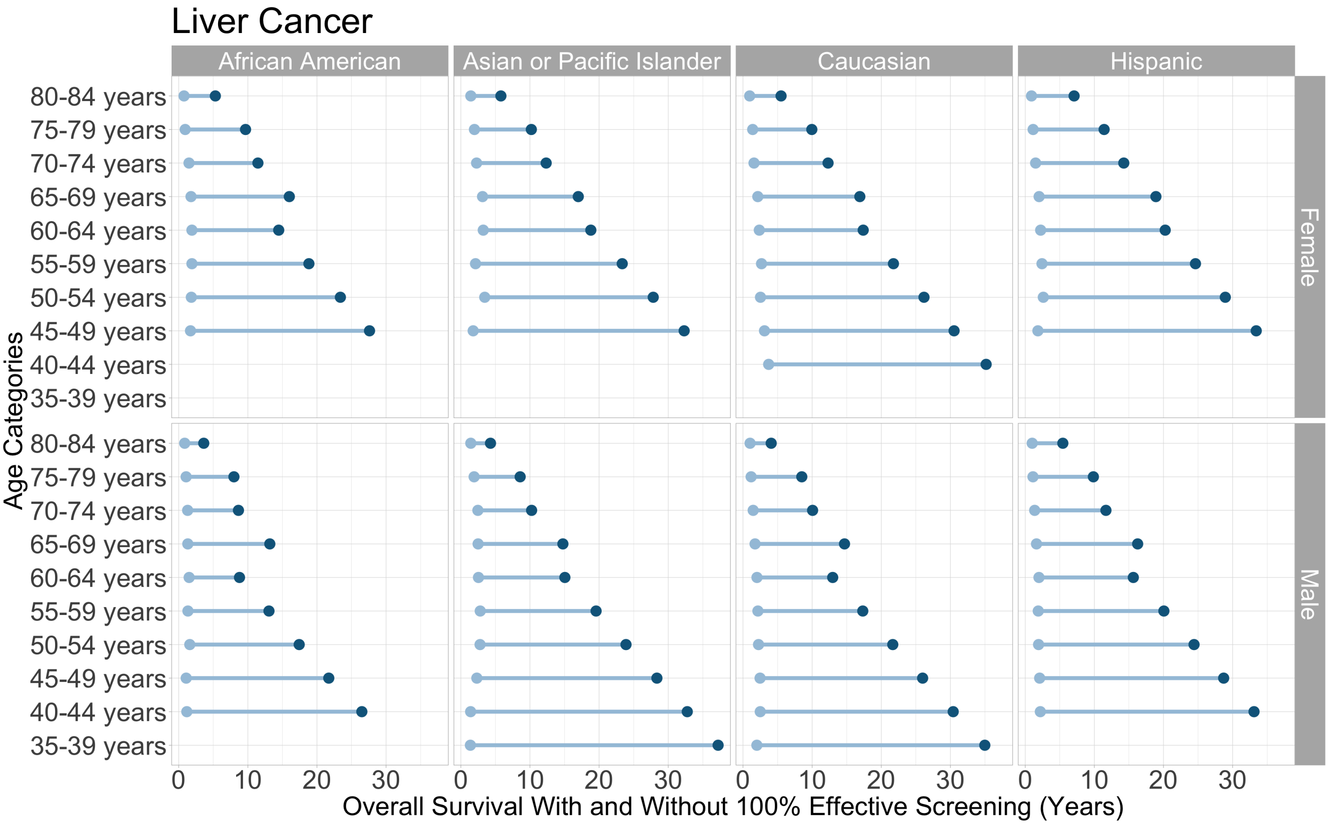

Fig G – Difference in overall survival between an effective screening program and no screening for liver cancer. The left end of the dumbbell shows expected survival of unscreened cancer sufferers, the right end shows estimated average survival under a screening program with 100% efficacy.

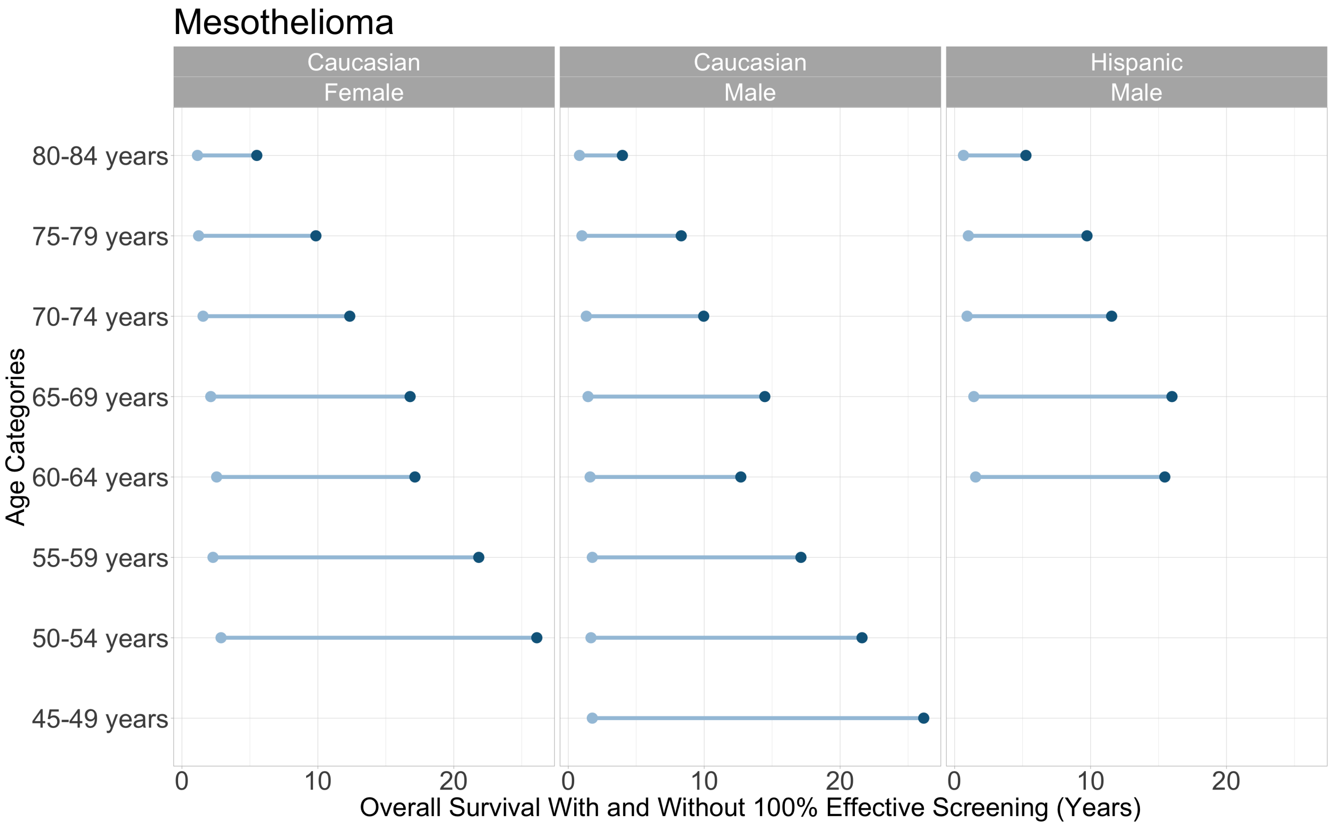

Fig H – Difference in overall survival between an effective screening program and no screening for mesothelioma. The left end of the dumbbell shows expected survival of unscreened cancer sufferers, the right end shows estimated average survival under a screening program with 100% efficacy. There was not enough data to make reliable estimates from many population subgroups.

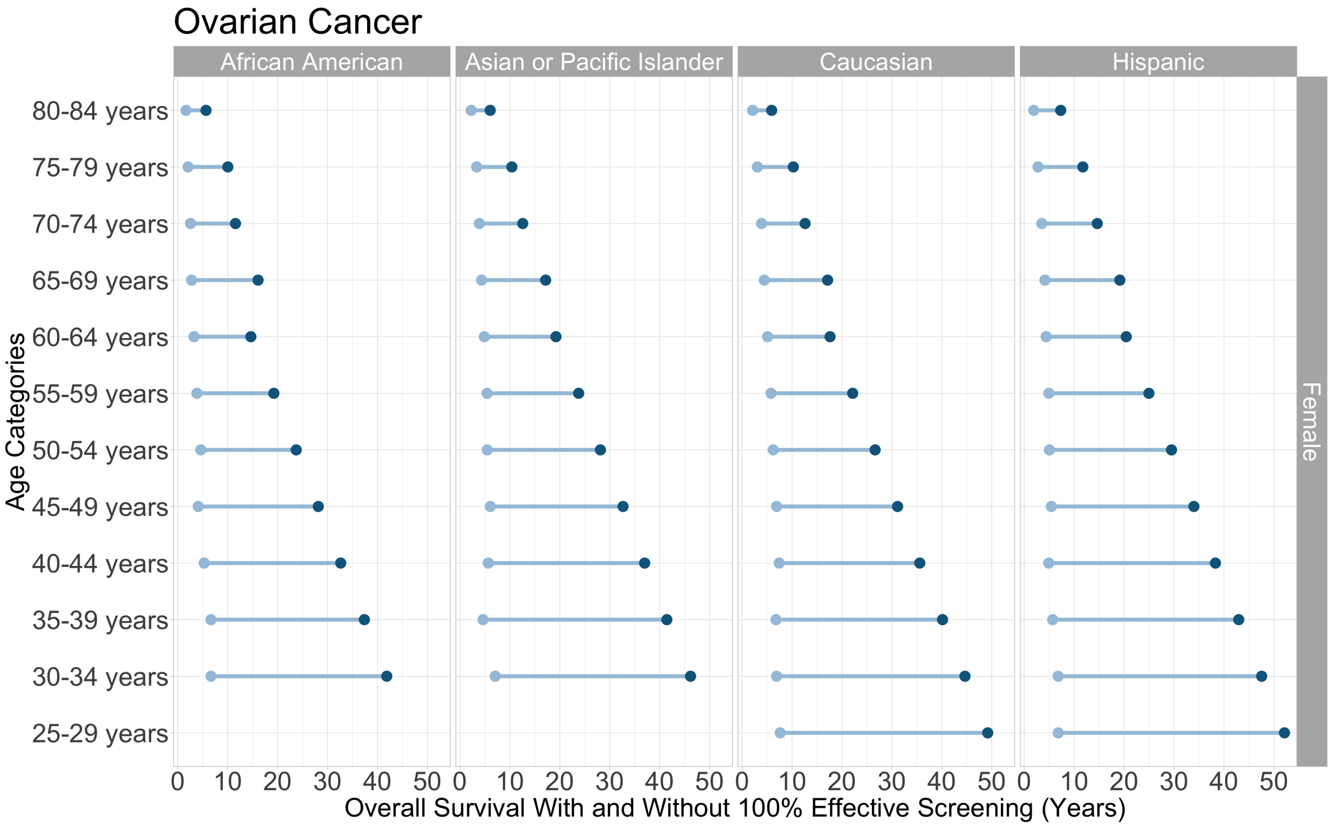

Fig I – Difference in overall survival between an effective screening program and no screening for ovarian cancer. The left end of the dumbbell shows expected survival of unscreened cancer sufferers, the right end shows estimated average survival under a screening program with 100% efficacy.

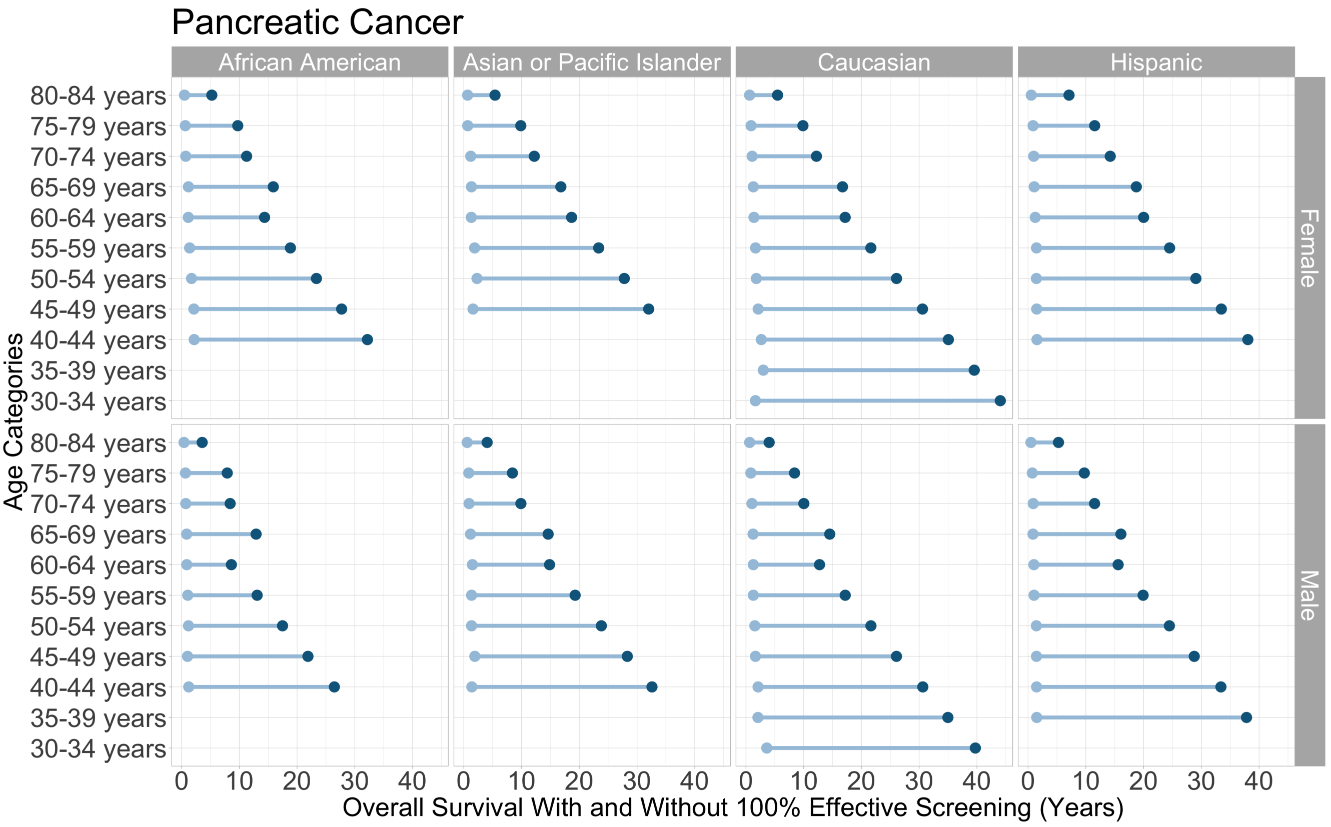

Fig J – Difference in overall survival between an effective screening program and no screening for pancreatic cancer. The left end of the dumbbell shows expected survival of unscreened cancer sufferers, the right end shows estimated average survival under a screening program with 100% efficacy.

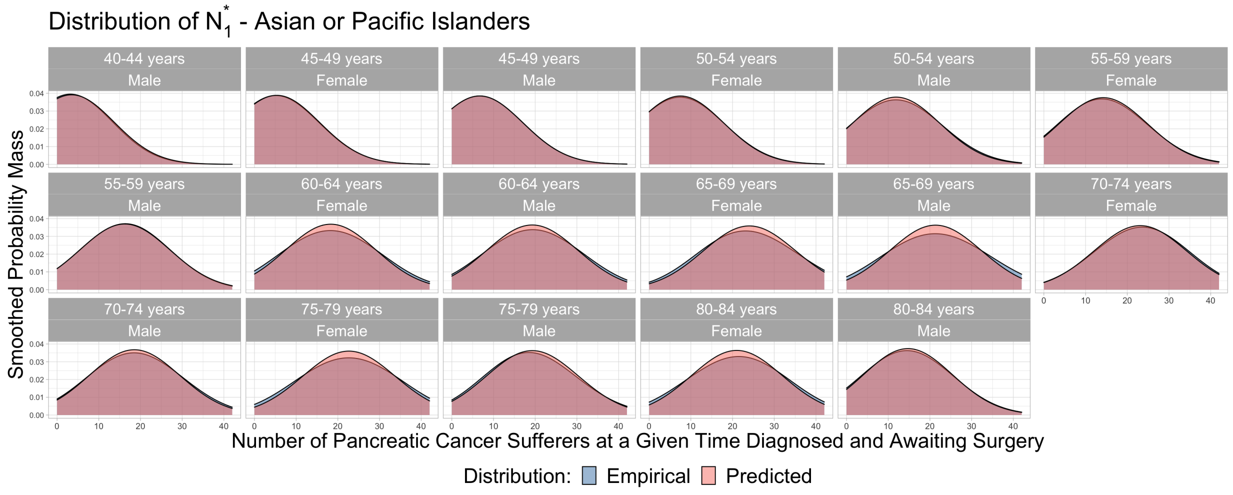

Fig K – Testing the distributional predictions of the BCMP theorem for number of newly diagnosed Asian or Pacific Islander pancreatic cancer sufferers. We compare the predicted distribution of N_1^*, the number of pancreatic cancer sufferers at a given time diagnosed and awaiting surgery, and the empirical distribution of this quantity estimated from the incidence data. The theorem predicts that N_1^* should have a Poisson distribution whose mean is found by solving the traffic equations. This process only involves averages of the data, and not details of the data itself. The empirical distribution is also a discrete distribution, but we smooth out the probability mass function of each as it is easier to visualize this way.

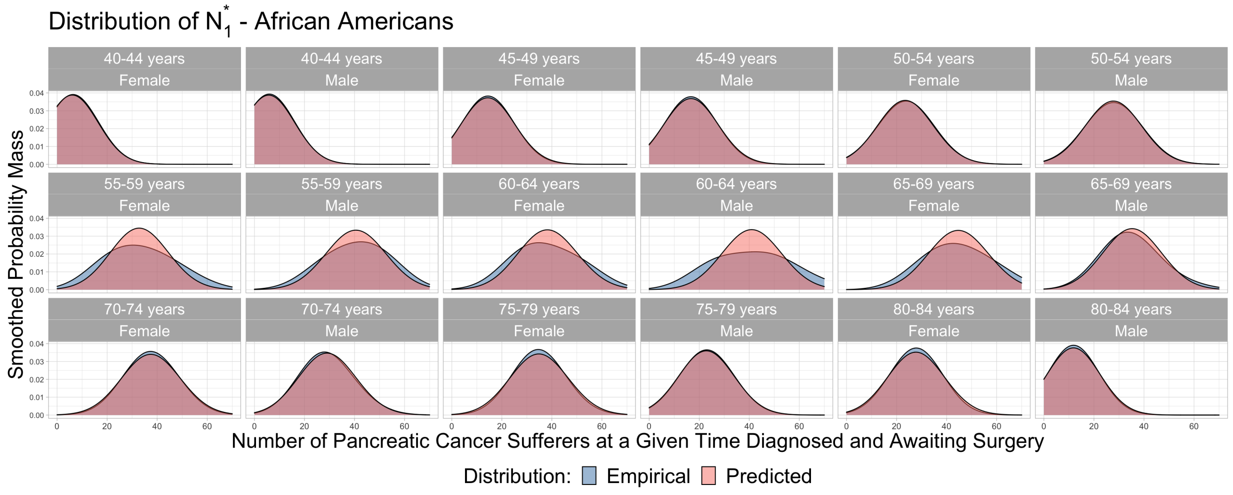

Fig L – Testing the distributional predictions of the BCMP theorem for number of newly diagnosed African American pancreatic cancer sufferers. We compare the predicted distribution of N_1^*, the number of pancreatic cancer sufferers at a given time diagnosed and awaiting surgery, and the empirical distribution of this quantity estimated from the incidence data. The theorem predicts that N_1^* should have a Poisson distribution whose mean is found by solving the traffic equations. This process only involves averages of the data, and not details of the data itself. The empirical distribution is also a discrete distribution, but we smooth out the probability mass function of each as it is easier to visualize this way.

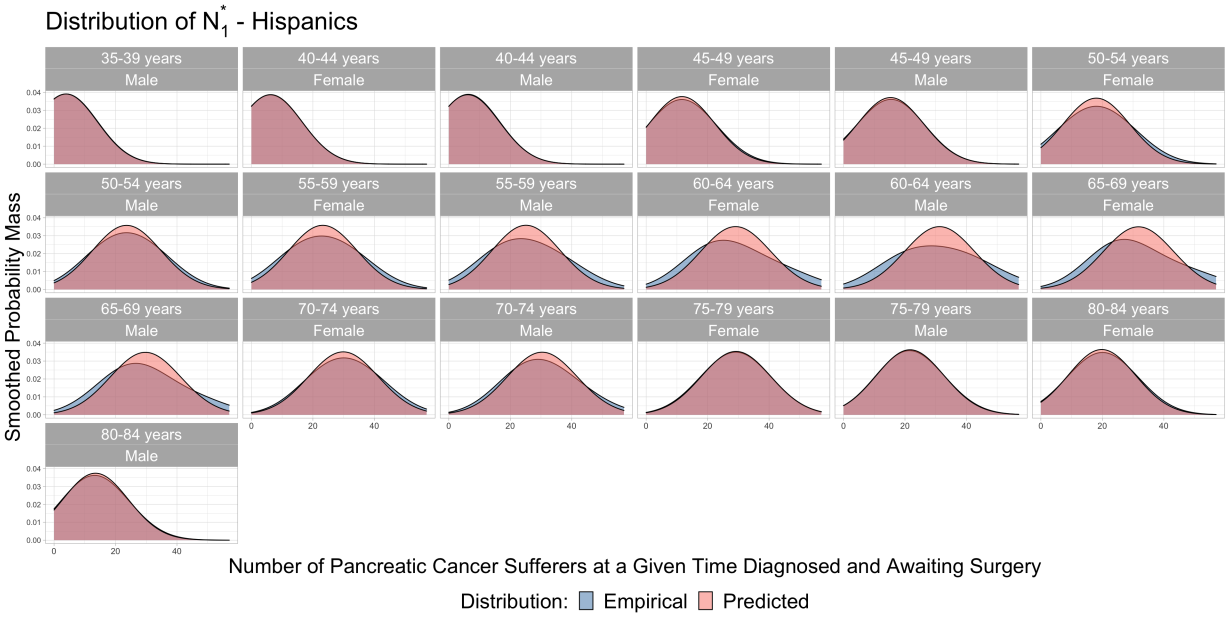

Fig M – Testing the distributional predictions of the BCMP theorem for number of newly diagnosed Hispanic pancreatic cancer sufferers. We compare the predicted distribution of N_1^*, the number of pancreatic cancer sufferers at a given time diagnosed and awaiting surgery, and the empirical distribution of this quantity estimated from the incidence data. The theorem predicts that N_1^* should have a Poisson distribution whose mean is found by solving the traffic equations. This process only involves averages of the data, and not details of the data itself. The empirical distribution is also a discrete distribution, but we smooth out the probability mass function of each as it is easier to visualize this way.

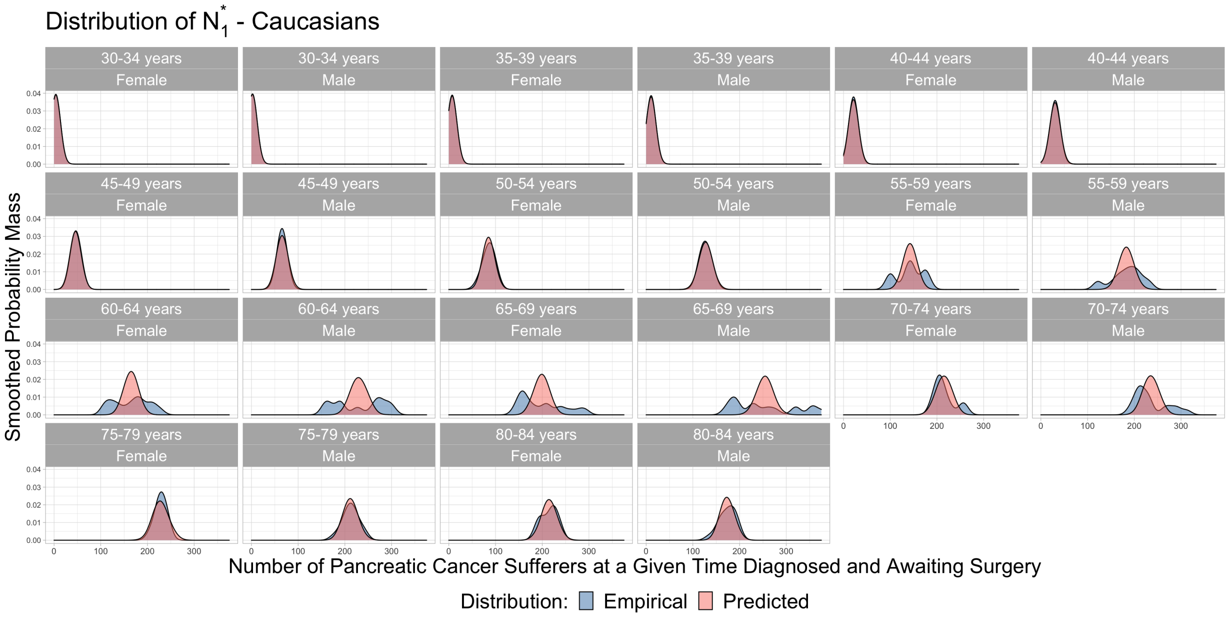

Fig N – Testing the distributional predictions of the BCMP theorem for number of newly diagnosed Caucasian pancreatic cancer sufferers. We compare the predicted distribution of N_1^*, the number of pancreatic cancer sufferers at a given time diagnosed and awaiting surgery, and the empirical distribution of this quantity estimated from the incidence data. The theorem predicts that N_1^* should have a Poisson distribution whose mean is found by solving the traffic equations. This process only involves averages of the data, and not details of the data itself. The empirical distribution is also a discrete distribution, but we smooth out the probability mass function of each as it is easier to visualize this way.

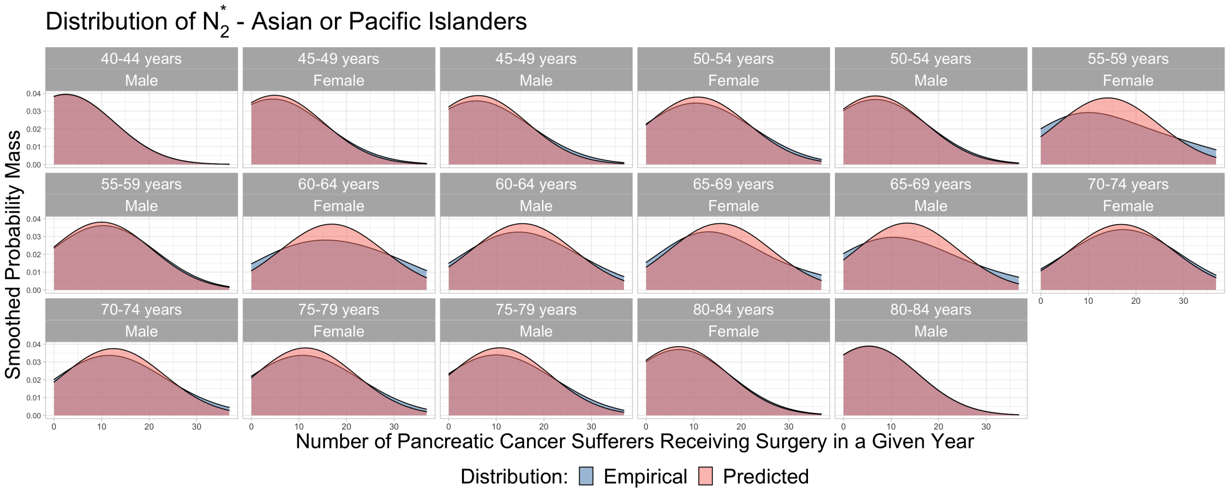

Fig O – Testing the distributional predictions of the BCMP theorem for Asian and Pacific Islander pancreatic cancer sufferers receiving surgery. We compare the predicted distribution of N_2^*, the number of pancreatic cancer sufferers receiving surgery in a given year, and the empirical distribution of this quantity estimated from the incidence data. The theorem predicts that N_2^* should have a Poisson distribution whose mean is found by solving the traffic equations. This process only involves averages of the data, and not details of the data itself. The empirical distribution is also a discrete distribution, but we smooth out the probability mass function of each as it is easier to visualize this way.

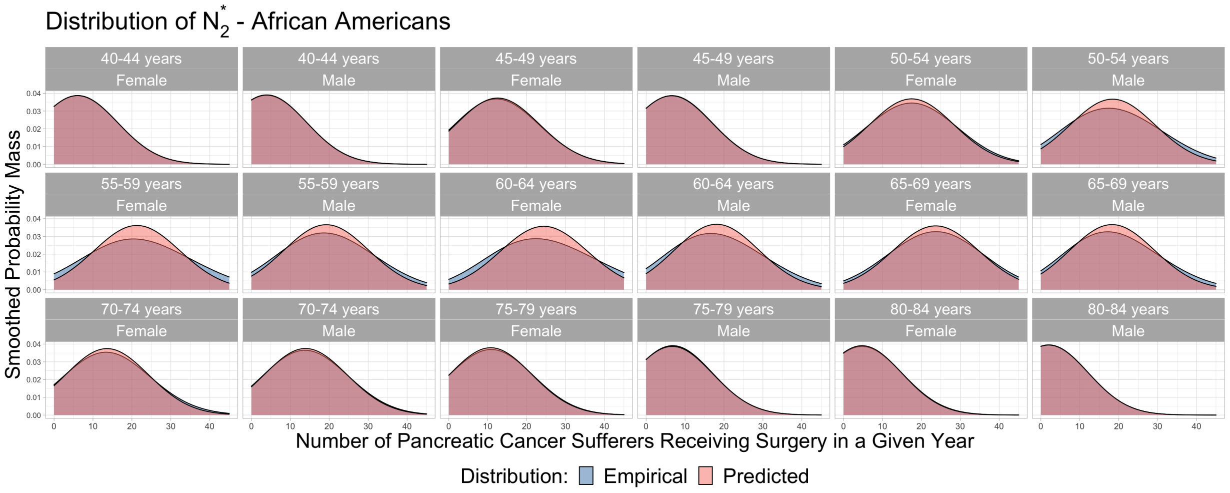

Fig P – Testing the distributional predictions of the BCMP theorem for African American pancreatic cancer sufferers receiving surgery. We compare the predicted distribution of N_2^*, the number of pancreatic cancer sufferers receiving surgery in a given year, and the empirical distribution of this quantity estimated from the incidence data. The theorem predicts that N_2^* should have a Poisson distribution whose mean is found by solving the traffic equations. This process only involves averages of the data, and not details of the data itself. The empirical distribution is also a discrete distribution, but we smooth out the probability mass function of each as it is easier to visualize this way.

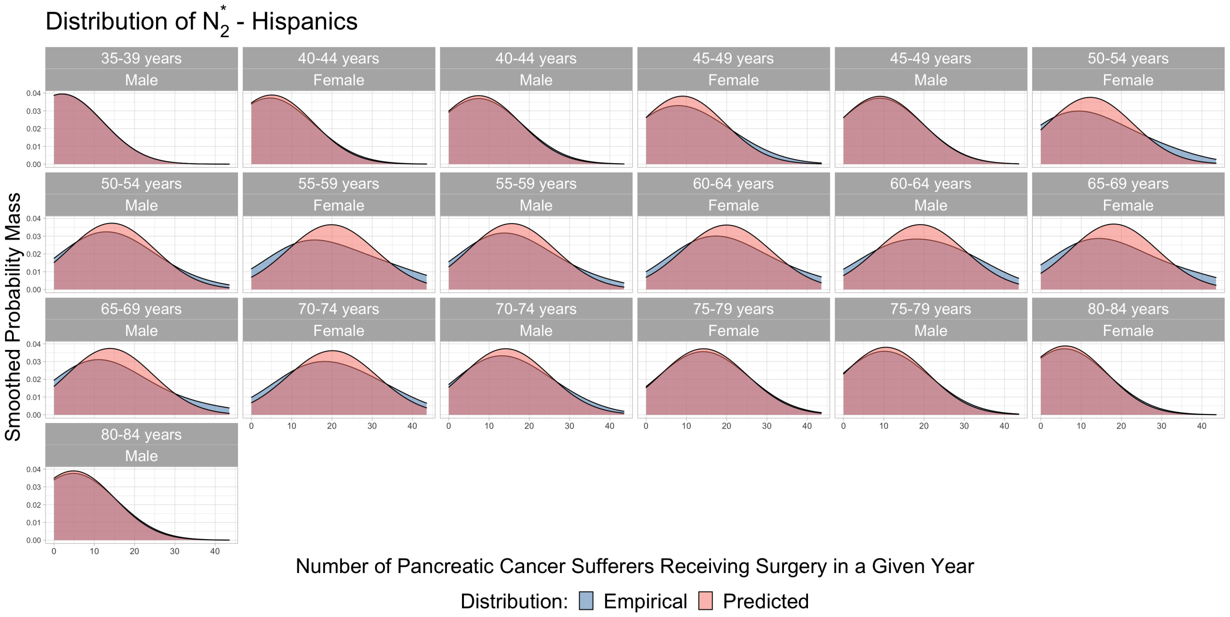

Fig Q – Testing the distributional predictions of the BCMP theorem for Hispanic pancreatic cancer sufferers receiving surgery. We compare the predicted distribution of N_2^*, the number of pancreatic cancer sufferers receiving surgery in a given year, and the empirical distribution of this quantity estimated from the incidence data. The theorem predicts that N_2^* should have a Poisson distribution whose mean is found by solving the traffic equations. This process only involves averages of the data, and not details of the data itself. The empirical distribution is also a discrete distribution, but we smooth out the probability mass function of each as it is easier to visualize this way.

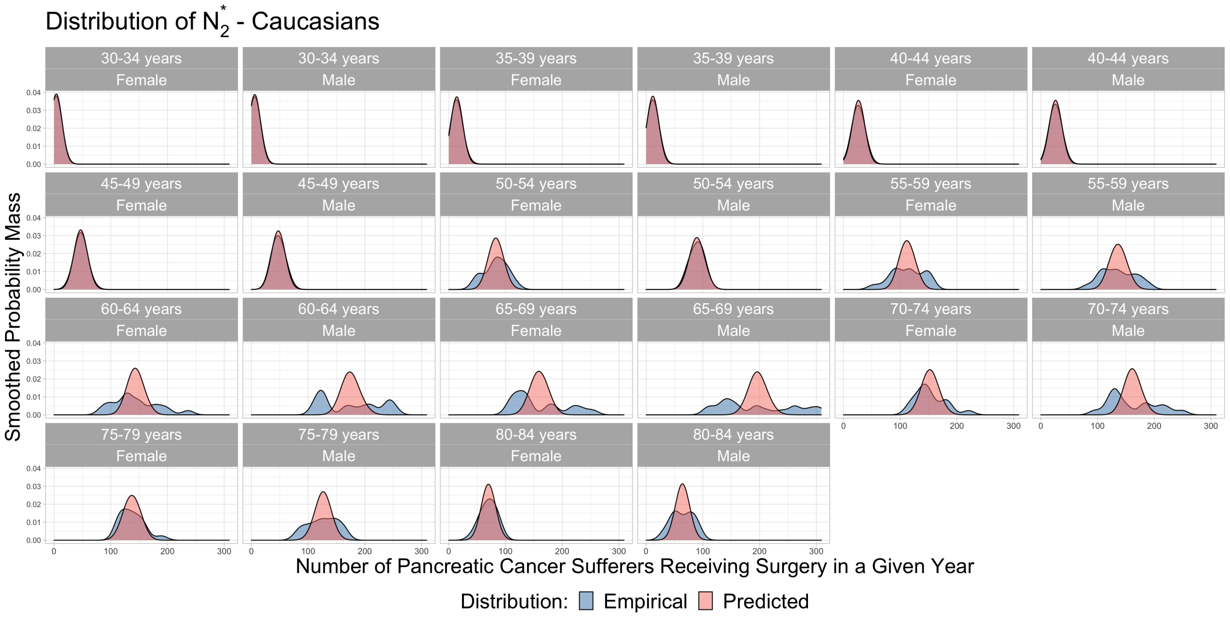

Fig R – Testing the distributional predictions of the BCMP theorem for Caucasian pancreatic cancer sufferers receiving surgery. We compare the predicted distribution of N_2^*, the number of pancreatic cancer sufferers receiving surgery in a given year, and the empirical distribution of this quantity estimated from the incidence data. The theorem predicts that N_2^* should have a Poisson distribution whose mean is found by solving the traffic equations. This process only involves averages of the data, and not details of the data itself. The empirical distribution is also a discrete distribution, but we smooth out the probability mass function of each as it is easier to visualize this way.

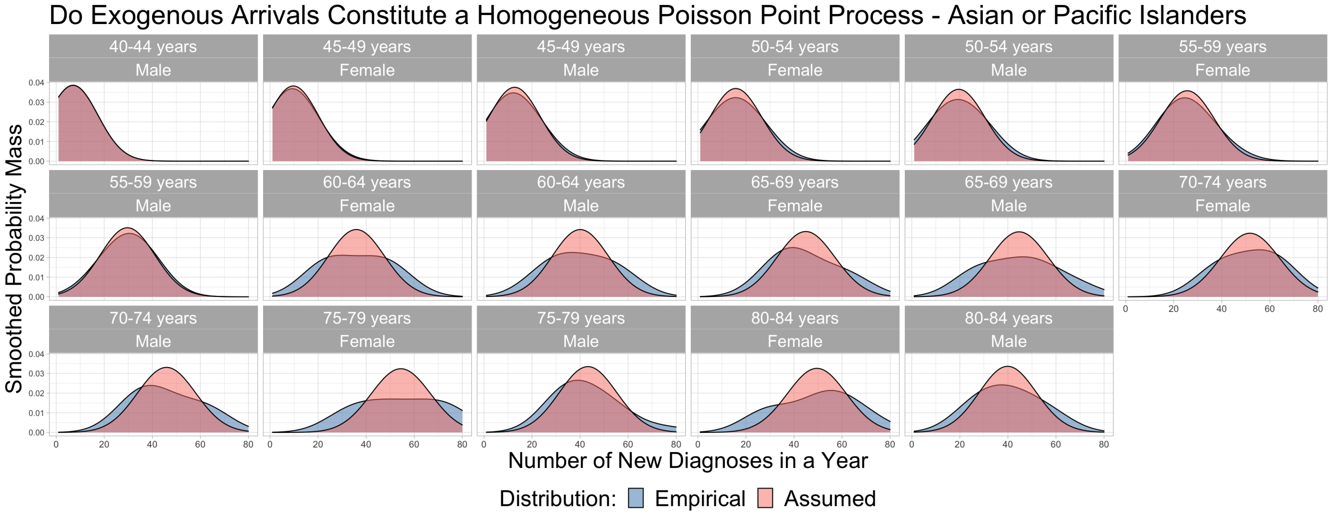

Fig S – Do pancreatic cancer diagnoses of Asian and Pacific Islanders follow the Markovian assumption for exogenous arrivals required by the BCMP theorem? We test the model assumption that exogenous arrivals constitute homogeneous Poisson point processes. We fix a time interval of length one year and then ask if the number of arrival increments during that period follow a Poisson distribution with a mean given by the estimated arrival rate multiplied by the time period, one year. This is necessary (but not sufficient) to be a Poisson point process, so if this is violated then certainly the assumption of Markovian arrivals is too. The Poisson distribution and the empirical distribution of the number of patients diagnosed each year are discrete distributions, but we smooth out their probability mass functions for ease of viewing and comparing. The assumed distribution comes solely from averages of the diagnosis data and does not use anything else about the data itself. The empirical distribution is a result of looking at incidence each year broken down by population subgroup.

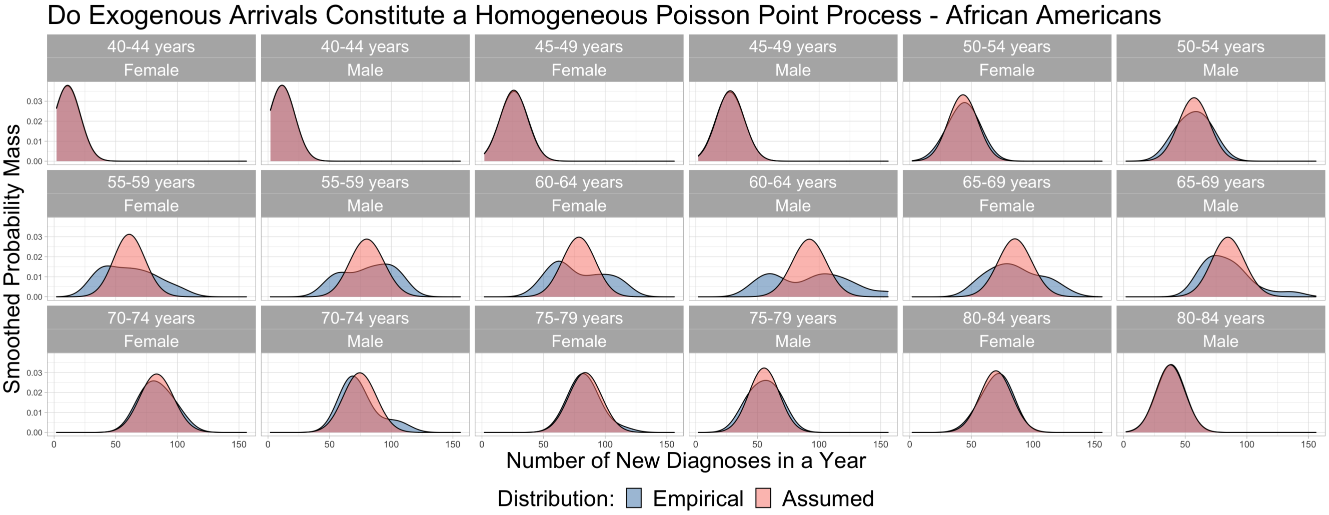

Fig T – Do pancreatic cancer diagnoses of African Americans follow the Markovian assumption for exogenous arrivals required by the BCMP theorem? We test the model assumption that exogenous arrivals constitute homogeneous Poisson point processes. We fix a time interval of length one year and then ask if the number of arrival increments during that period follow a Poisson distribution with a mean given by the estimated arrival rate multiplied by the time period, one year. This is necessary (but not sufficient) to be a Poisson point process, so if this is violated then certainly the assumption of Markovian arrivals is too. The Poisson distribution and the empirical distribution of the number of patients diagnosed each year are discrete distributions, but we smooth out their probability mass functions for ease of viewing and comparing. The assumed distribution comes solely from averages of the diagnosis data and does not use anything else about the data itself. The empirical distribution is a result of looking at incidence each year broken down by population subgroup.

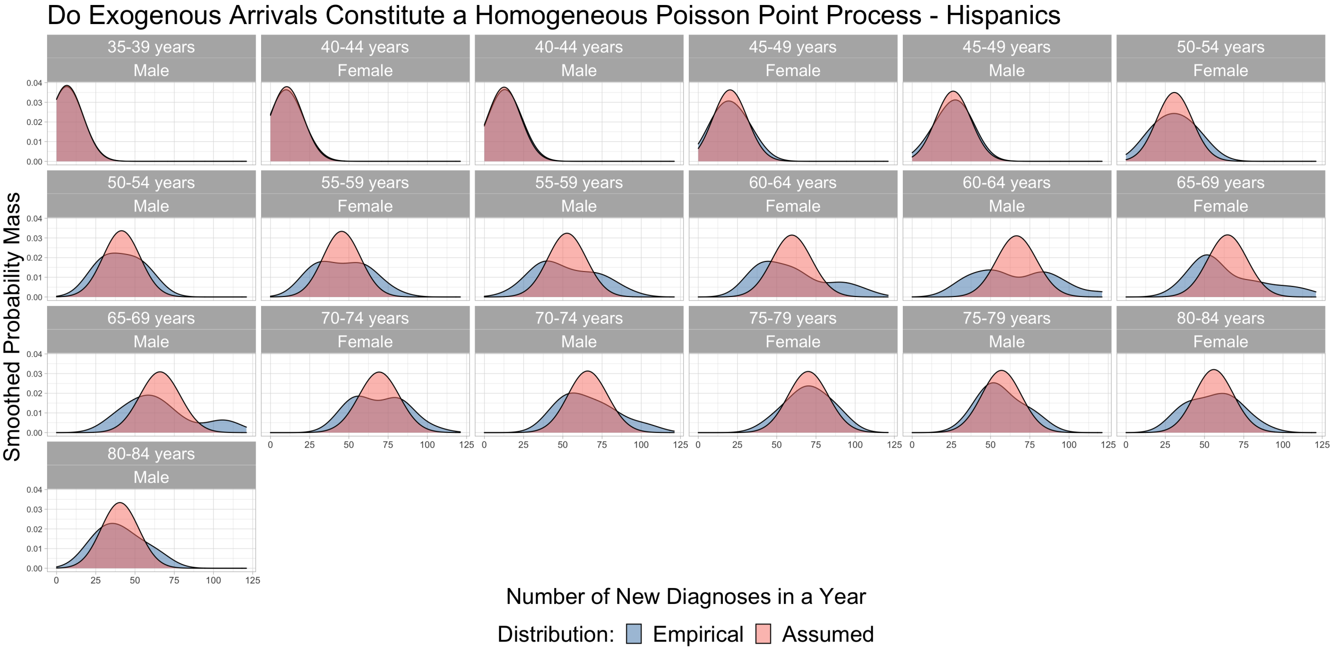

Fig U – Do pancreatic cancer diagnoses of Hispanics follow the Markovian assumption for exogenous arrivals required by the BCMP theorem? We test the model assumption that exogenous arrivals constitute homogeneous Poisson point processes. We fix a time interval of length one year and then ask if the number of arrival increments during that period follow a Poisson distribution with a mean given by the estimated arrival rate multiplied by the time period, one year. This is necessary (but not sufficient) to be a Poisson point process, so if this is violated then certainly the assumption of Markovian arrivals is too. The Poisson distribution and the empirical distribution of the number of patients diagnosed each year are discrete distributions, but we smooth out their probability mass functions for ease of viewing and comparing. The assumed distribution comes solely from averages of the diagnosis data and does not use anything else about the data itself. The empirical distribution is a result of looking at incidence each year broken down by population subgroup.

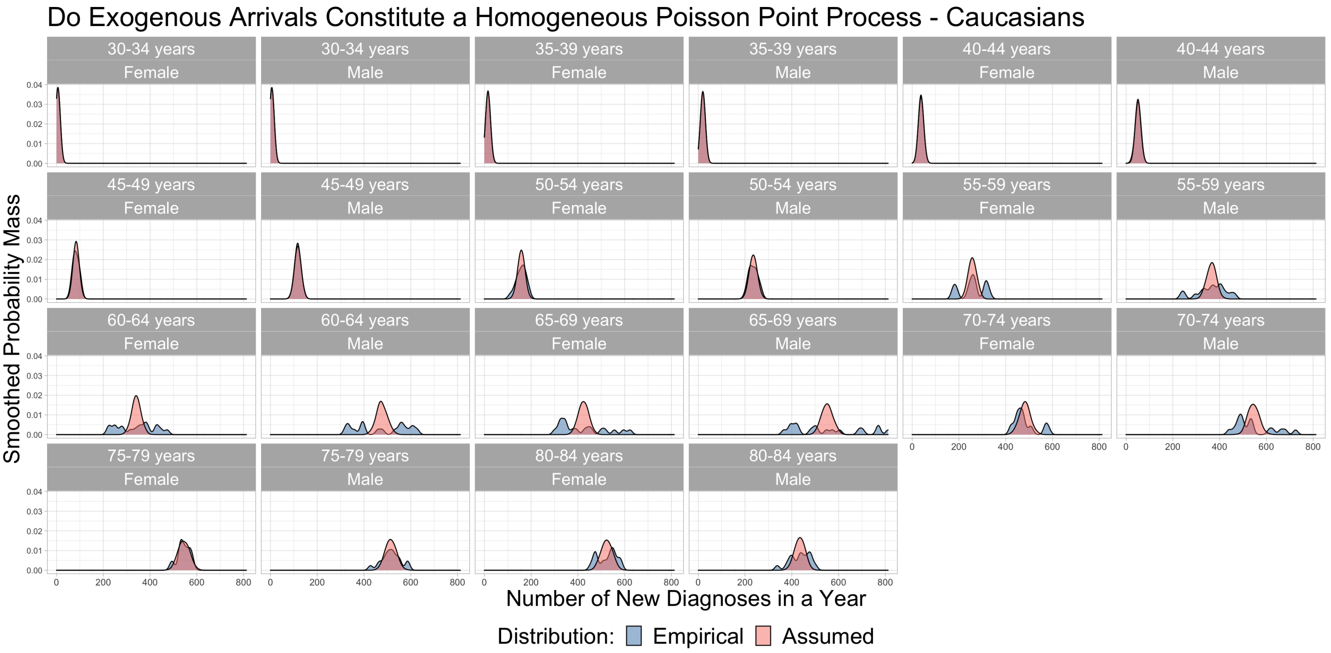

Fig V – Do pancreatic cancer diagnoses of Caucasians follow the Markovian assumption for exogenous arrivals required by the BCMP theorem? We test the model assumption that exogenous arrivals constitute homogeneous Poisson point processes. We fix a time interval of length one year and then ask if the number of arrival increments during that period follow a Poisson distribution with a mean given by the estimated arrival rate multiplied by the time period, one year. This is necessary (but not sufficient) to be a Poisson point process, so if this is violated then certainly the assumption of Markovian arrivals is too. The Poisson distribution and the empirical distribution of the number of patients diagnosed each year are discrete distributions, but we smooth out their probability mass functions for ease of viewing and comparing. The assumed distribution comes solely from averages of the diagnosis data and does not use anything else about the data itself. The empirical distribution is a result of looking at incidence each year broken down by population subgroup.

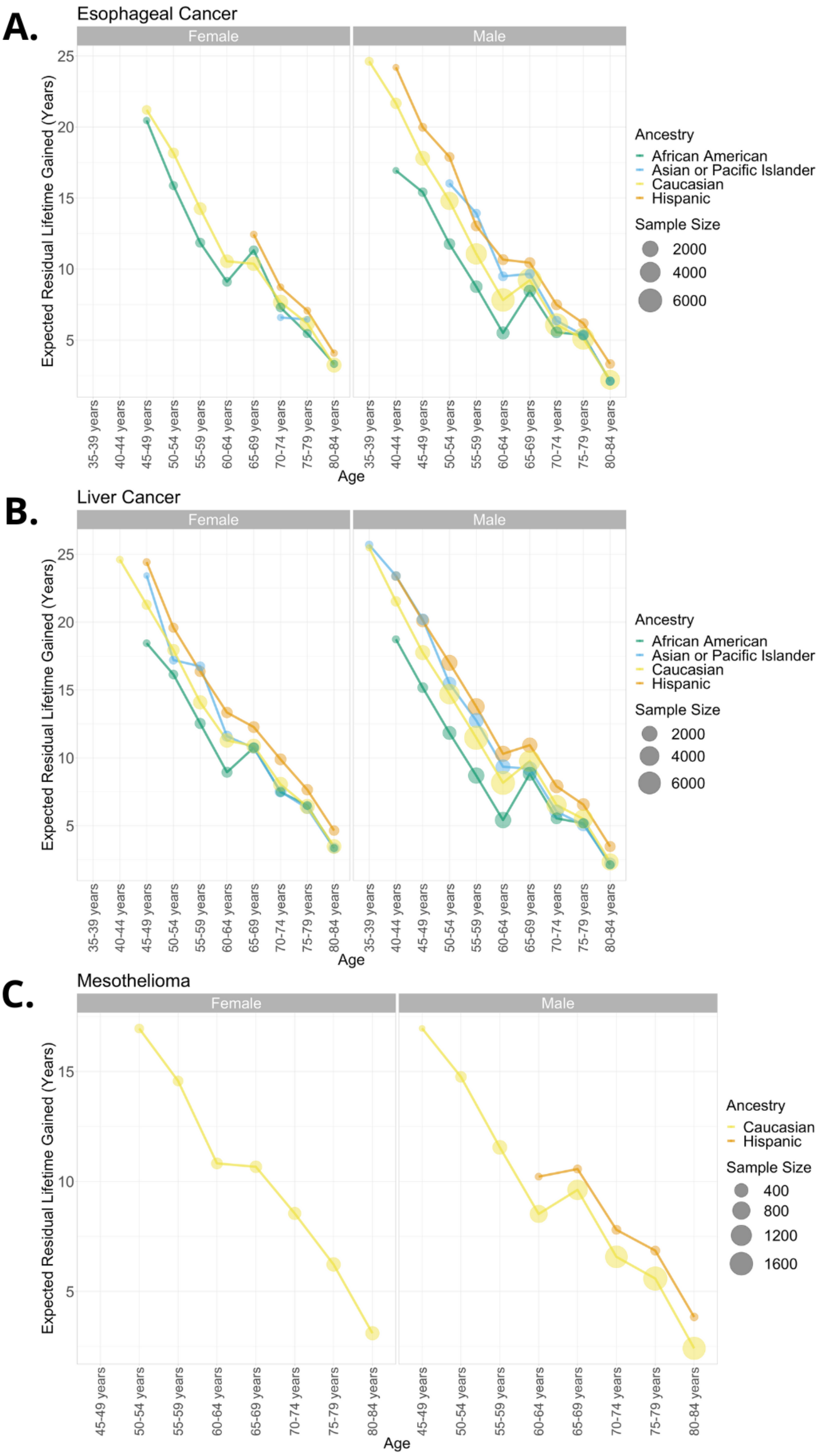

Fig W – Per patient expected lifetime gain of putative screening programs for a variety of cancer types for which there is currently no widespread screening. (A) Overall predicted survival benefit from a potential esophageal cancer screen of 75% effectiveness stratified by age, sex and ancestry. There is an artificial bump at age 65 due to the discrete stratification of conditional lifetime distributions. (B) Analogous plot for a potential liver cancer screen under the same assumptions. The sample size used to estimate the parameters is given by the point size and no estimate is made where the sample size is too small to be reliable. (C) Analogous plot for mesothelioma screening under the same assumptions.

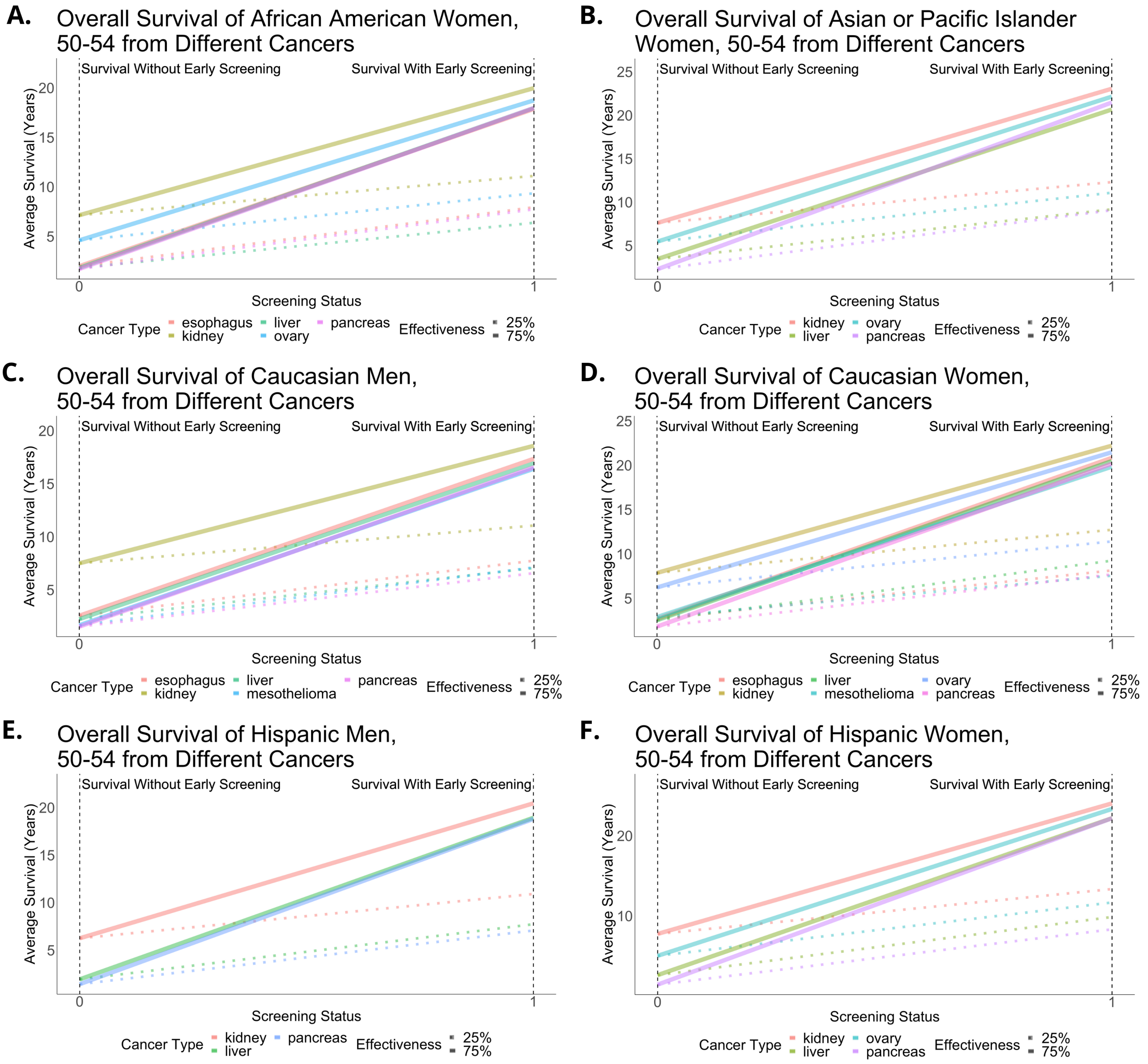

Fig X – Comparison of the change in overall survival as a result of screening for different cancer types for various population subgroups aged 50-54. Survival without screening is shown on the left-hand side and the predicted survival with screening is on the right. We display two scenarios: one in which screening has an effectiveness of 25% and another with effectiveness of 75%.
